# AAPH-induced oxidative damage reduced anion exchanger 1 (SLC4A1/AE1) activity in human red blood cells: protective effect of an anthocyanin-rich extract

**DOI:** 10.3389/fphys.2023.1303815

**Published:** 2023-12-04

**Authors:** Alessia Remigante, Sara Spinelli, Giuseppe Tancredi Patanè, Davide Barreca, Elisabetta Straface, Lucrezia Gambardella, Giuseppina Bozzuto, Daniele Caruso, Giuseppe Falliti, Silvia Dossena, Angela Marino, Rossana Morabito

**Affiliations:** ^1^ Department of Chemical Biological, Pharmaceutical and Environmental Sciences, University of Messina, Messina, Italy; ^2^ Biomarkers Unit, Center for Gender-Specific Medicine, Istituto Superiore di Sanità, Rome, Italy; ^3^ National Center for Drug Research and Evaluation, Istituto Superiore di Sanità, Rome, Italy; ^4^ Complex Operational Unit of Clinical Pathology of Papardo Hospital, Messina, Italy; ^5^ Institute of Pharmacology and Toxicology, Paracelsus Medical University, Salzburg, Austria

**Keywords:** oxidative stress, erythrocytes, anthocyanins, anion, exchange, methemoglobin

## Abstract

**Introduction:** During their lifespan in the bloodstream, red blood cells (RBCs) are exposed to multiple stressors, including increased oxidative stress, which can affect their morphology and function, thereby contributing to disease.

**Aim:** This investigation aimed to explore the cellular and molecular mechanisms related to oxidative stress underlying anion exchanger 1 activity (band 3, SLC4A1/AE1) in human RBCs. To achieve this aim, the relationship between RBC morphology and functional and metabolic activity has been explored. Moreover, the potential protective effect of an anthocyanin-enriched fraction extracted from *Callistemon citrinus* flowers was studied.

**Methods:** Cellular morphology, parameters of oxidative stress, as well as the anion exchange capability of band 3 have been analyzed in RBCs treated for 1 h with 50 mM of the pro-oxidant 2,2′-azobis (2-methylpropionamide)-dihydrochloride (AAPH). Before or after the oxidative insult, subsets of cells were exposed to 0.01 μg/mL of an anthocyanin-enriched fraction for 1 h.

**Results:** Exposure to AAPH caused oxidative stress, exhaustion of reduced glutathione, and over-activation of the endogenous antioxidant machinery, resulting in morphological alterations of RBCs, specifically the formation of acanthocytes, increased lipid peroxidation and oxidation of proteins, as well as abnormal distribution and hyper-phosphorylation of band 3. Expected, oxidative stress was also associated with a decreased band 3 ion transport activity and an increase of oxidized haemoglobin, which led to abnormal clustering of band 3. Exposure of cells to the anthocyanin-enriched fraction prior to, but not after, oxidative stress efficiently counteracted oxidative stress-related alterations. Importantly, protection of band3 function from oxidative stress could only be achieved in intact cells and not in RBC ghosts.

**Conclusion:** These findings contribute a) to clarify oxidative stress-related physiological and biochemical alterations in human RBCs, b) propose anthocyanins as natural antioxidants to neutralize oxidative stress-related modifications, and 3) suggest that cell integrity, and therefore a cytosolic component, is required to reverse oxidative stress-related pathophysiological derangements in human mature RBCs.

## 1 Introduction

Human red blood cells (RBC) have maximized their functional capacity to transport and deliver oxygen to tissues and cells of the body through the progressive loss of the cytoplasmatic organelles, including nuclei, which occurs during their maturation process from erythroid precursors to reticulocytes, and ultimately, to mature biconcave RBCs (Moras et al., 2017). As a result, each mature RBC encloses about 250–270 hemoglobin million copies, which account for 98% of the cytosolic proteome (D'Alessandro and Zolla, 2017). At full oxygen saturation, mature RBCs can carry up to 1 billion molecules of oxygen/cell (Mohanty et al., 2014). Therefore, the major endogenous source of intracellular reactive species in RBCs is the slow auto-oxidation of haemoglobin, which produces non-functional methaemoglobin and superoxide radicals that rapidly dismutase to form hydrogen peroxide (H_2_O_2_) (Fujii et al., 2021). In addition, exogenous reactive molecules can be released in the bloodstream by macrophages, neutrophils, and endothelial cells (Pretini et al., 2019). Oxidative damage to any of the biological macromolecules (Akki et al., 2018; Akki et al., 2022) of RBCs can affect the integrity and stability of their cellular structure and their functional and metabolic activity (Xiong et al., 2013; Vona et al., 2019; D'Alessandro et al., 2021). Lipid peroxidation is one of the most damaging reactions of free radicals in RBCs. It is well documented that lipid peroxidation is the outcome of the oxidation of membrane polyunsaturated fatty acids (PUFA), which interferes with the canonical structure and physiological functions of the RBC plasma membrane. These oxidative events generate hydroperoxides and secondary products that ultimately result in structural disruption of cell-surface lipid bilayer and protein carbonylation and/or protein thiol oxidation of plasma membrane-bound proteins (Pandey and Rizvi, 2011; Contreras-Puentes et al., 2019; Zhang et al., 2019; Amezaga et al., 2020; Akki et al., 2022; Remigante and Morabito, 2022).

Since RBCs are larger than the diameter of capillaries in the micro-circulation, they have to deform and squeeze into blood capillaries to deliver oxygen to the cells and tissues of the body as needed (Ebrahimi and Bagchi, 2022). In this context, oxidative damage to the plasma membrane as well as cytoskeletal proteins impairs the rheologic properties of circulating RBCs (Mazzulla et al., 2015). These abnormalities include i) decreased deformability, ii) increased membrane micro-viscosity, and iii) increased RBC aggregation (Maruyama et al., 2022). There is a number of investigations showing that injury at the level of the plasma membrane and cytoskeletal proteins impairs RBC deformability. To name just a few examples, Jiang and co-authors proved that increased spectrin glycosylation induced by augmented intracellular oxidative stress condition led to deformability abnormalities in rat diabetic RBCs, which is not surprising, as diabetes generates oxygen free radicals (Jiang et al., 1990). Additionally, a recent study performed by Spinelli and collaborators demonstrated that oxidative stress related to natural aging impaired human RBCs deformability, resulting in structural rearrangements of the membrane cytoskeleton-associated proteins spectrin, ankyrin, and protein 4.1, and increased tyrosine phosphorylation of plasma membrane-bound band 3 protein (Vallese et al., 2022). Band 3 (SLC4A1/AE1) (Remigante et al., 2022b), the most abundant RBC membrane protein, possesses two different cytosolic domains (Arakawa et al., 2015). At high oxygen saturation, the N-terminal domain can serve as an inhibitory docking site for glycolytic enzymes. On the contrary, at low oxygen saturation, deoxyhemoglobin competes with the glycolytic enzymes and displaces them from the plasma membrane to boost glycolysis and stimulate both ATP and 2,3-diphosphoglycerate production. Prolonged oxidant stress, for example, during RBC storage in blood banks, triggers proteolysis of the band 3 N-terminal domain, thus provoking the loss of this RBC oxygen-dependent metabolic modulation pathway (Mandal et al., 2003; Rinalducci et al., 2012; Matte et al., 2013). Moreover, oxidative stress-related events are well known to cross-link band 3 dimers and tetramers and, also, band 3 and haemoglobin, thus leading to the formation of high molecular mass aggregates (Rinalducci et al., 2012). On the other hand, the C-terminal domain contains the anion-transport functional region that mediates the chloride-bicarbonate exchange across the RBC membrane (Reithmeier et al., 2016). Membrane ionic transport systems, including band 3, are involved in the maintenance of cellular homeostasis during exposure to stressors and are therefore accurately regulated to respond to stress conditions (Remigante et al., 2021a).

To cope with the oxidative stress effects, RBCs have developed extensive endogenous antioxidant machinery involving both non-enzymatic antioxidants, such as glutathione, and enzymatic antioxidants, including catalase, superoxide dismutase, peroxiredoxin-2, and glutathione peroxidase (Martemucci et al., 2022). Also, natural secondary metabolites with antioxidant properties might play a protective function in this context. Multiple polyphenol-rich extracts of plant origin have been proven as an excellent and workable alternative for supporting intracellular antioxidant defense during elevated oxidative stress owing to their activity as ROS scavengers and/or inhibitors (Niedzwiecki et al., 2016; Kouka et al., 2017; Philip et al., 2019; Luo et al., 2021; Monjotin et al., 2022). By definition, antioxidants are molecules capable of inhibiting and/or quenching free radical reactions in order to delay or prevent cellular injury. In this context, an anthocyanin-enriched fraction extracted from *Callistemon citrinus* (Curtis, skeels), which is an ornamental and medicinal plant from the *Myrtaceae* family, showed antioxidant power *in vitro* and in cell-based assays (Lagana et al., 2020). Anthocyanins are water-soluble pigments belonging to the polyphenol class of compounds and are responsible for conferring red colors to flowers. The study of the constituents of therapeutic plants is of great value and essential for identifying novel antioxidants (Larayetan et al., 2017; Maury et al., 2020; Nishikito et al., 2023). However, the molecular mechanisms underlying the antioxidant action of polyphenol compounds in human RBCs have not yet been fully clarified and are still a matter of considerable debate.

**FIGURE 1:**
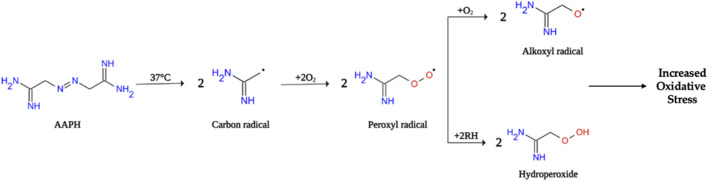
Schematic representation of AAPH decomposition in human RBCs. Inside the cell, the decomposition of AAPH at physiological temperature (37°C) generates free radicals (peroxyl radicals, alkoxyl radicals and hydroperoxide) that can attack the RBC membrane and induce lipid peroxidation leading to cell hemolysis. However, the generated radicals remain underneath the membrane bilayer then causing, oxidation of oxygenated hemoglobin to methemoglobin (Zimowska et al., 1997).

Among the various cell-based models of oxidative stress, 2,2′-azobis (2-methylpropionamide)-dihydrochloride (AAPH)-stimulated RBCs represent a useful system to study oxidative stress-related pathological states affecting the integrity of human RBC and leading to multi-organ dysfunction, such as haemolytic anemia, vaso-occlusion, chronic inflammation, and tissue damage (Zheng et al., 2016) (Figure 1). Thus, the present investigation aimed to explore the cellular and molecular mechanisms related to oxidative stress underlying anion exchanger 1 (band 3) activity in human RBCs. To achieve this aim, the relationship between RBC morphology, integrity, and functional activity has been explored. Moreover, the potential protective effect of an anthocyanin-enriched fraction extracted from *Callistemon citrinus* flowers was studied.

## 2 Results

### 2.1 Determination and quantification of phenolic compounds

An acidified ethanolic extract from *Callistemon citrinus* flowers was submitted to reverse phase high performance liquid chromatography coupled with diode array detection (RP-HPLC- DAD). The compounds found in the anthocyanin-enriched fraction are reported in Table 1. The inspection of UV spectra recorded between 200 and 800 nm, and simultaneous detection by diode array performed at 260, 290, 330, 370, and 520 nm only showed the presence of anthocyanin compounds in significant amounts, which was in agreement with literature data previously published by our group (Lagana et al., 2020), in particular: cyanidin-3,5-*O*-diglucoside (cyanin, 1); peonidin-3,5-*O*-diglucoside (peonin, 2); cyanidin-3-O-glucoside (3) and cyanidin-coumaroylglucoside-pyruvic acid (4). The most abundant compound was, by far, cyanidin-3,5-*O*-diglucoside (305.13 ± 2.82 mg/100 g DE) followed by peonidin-3,5-*O*-diglucoside and cyanidin-3-*O*-glucoside (172.06 ± 1.10 and 43.07 ± 0.61 mg/100 g DE, respectively), while the last compound (cyanidin-coumaroylglucoside-pyruvic acid) was present in significantly lower amount with respect to the other derivatives (9.75 ± 0.17 mg/100 g DE). Peak identity was confirmed by comparing their retention times and absorption spectra with those of pure (≥99%) commercially available standards and comparison with literature data.

**TABLE 1:** Identification and quantification of anthocyanin profile of the anthocyanin-enriched fraction by RP-HPLC-DAD analysis. Results are expressed as mean ± SD of three independent experiments (n = 3), and are quantified as mg cyanidin-3-*O*-glucoside equivalents (CyG) per 100 gr of dry extract (DE).

PEAK	Compound	Rt (min)	mg CyG/100 g DE
1	Cyanidin 3,5-*O*-diglucoside	17.8	305.13 ± 2.82
2	Peonidin-3,5-*O*-diglucoside	20.3	172.06 ± 1.10
3	Cyanidin-3-*O*-glucoside	22.5	43.07 ± 0.61
4	Cyanidin-coumaroylglucoside-pyruvic acid	25.3	9.75 ± 0.17

### 2.2 Anthocyanin-enriched fraction prevents cell shape modifications in AAPH-Incubated RBCs

In the present investigation, the first step was to assay a broad range of concentrations (from 0.01 μg/mL to 100 μg/mL) of an anthocyanin-enriched fraction as well as different incubation time intervals (30 min, 1 h, and 2 h), to exclude any damage in terms of haemolysis, lipid peroxidation, and protein oxidation, including the production of MetHb, which could be potentially provoked by direct exposure of RBCs to the extract (Supplementary Figure S1). This experimental procedure points to the importance of carefully establishing the proper concentration and incubation time for testing novel potential antioxidant compounds in cell-based assays. Based on these considerations, we selected a 1 h pre- and post-treatment with 0.01 μg/mL anthocyanin-enriched fraction, in order to estimate its potential antioxidant capacity.

**FIGURE 2:**
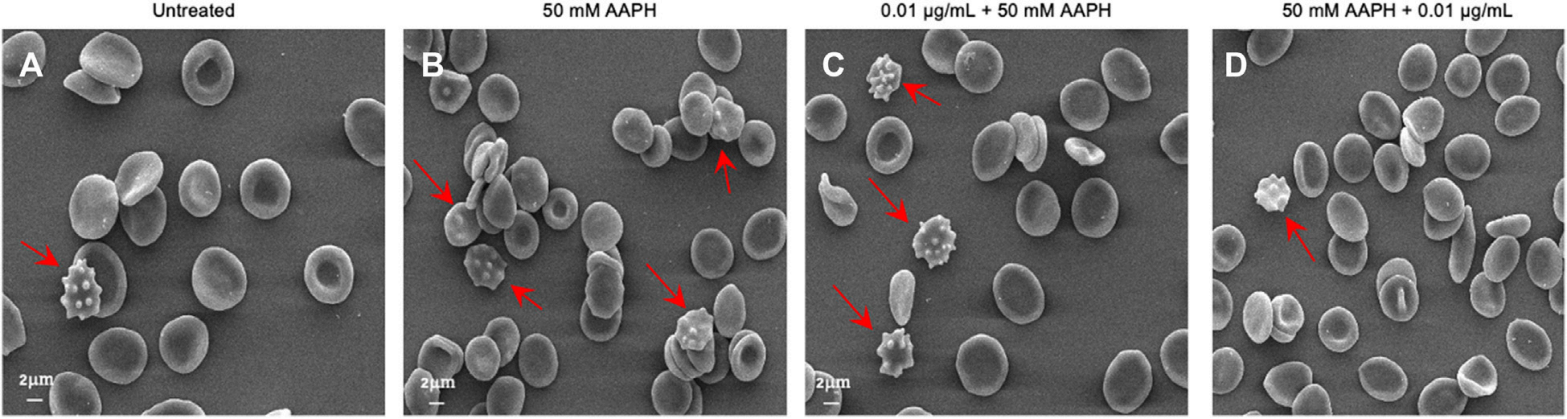
Evaluation of RBC morphology. Representative SEM images showing human RBCs with a canonical biconcave shape **(A)** untreated cells); **(B)** acanthocytes (arrows) after treatment with 50 mM AAPH (for 1 h, at 37°C); **(C)**. Pre-incubation with anthocyanin-enriched fraction (0.01 μg/mL for 1 h, at 37°C) still showed a remarkable cell morphology modification. On the contrary, **(D)** post-treatment with anthocyanin-enriched fraction (0.01 μg/mL for 1 h, at 37°C) attenuated the morphological changes compared to 50 mM AAPH exposure. Magnification 1,500×.

Based on these results, as shown in Figure 2, treatment for 1 h at 37°C with 50 mM AAPH provoked morphological changes in human RBCs. Indeed, in these experimental conditions, 23% of acanthocytes (surface blebs) were reported by scanning electron microscopy analysis. However, in samples pre-incubated with 0.01 μg/mL anthocyanin-enriched fraction (for 1 h, at 37°C) and then treated with 50 mM AAPH (1 h, at 37°C), the percentage of morphologically modified cells was reduced to 21%. Therefore, the generation of acanthocytes was not completely avoided by the pre-treatment with the anthocyanin-enriched fraction (Table 2). On the contrary, in human RBCs incubated with 50 mM AAPH and then exposed to 0.01 μg/mL anthocyanin-enriched fraction, we only noticed 9% of acanthocytes.

**TABLE 2:** Percentage of morphological alterations in RBCs left untreated or treated as reported. Data are presented as means ± S.E.M. from three independent experiments, where ns, not statistically significant *versus* untreated and 50 mM AAPH; ***p < 0.001 *versus* untreated; *p* < 0.001 *versus* 50 mM AAPH, one-way ANOVA followed by Bonferroni’s multiple comparison post-hoc test.

Experimental conditions	Acanthocytes
Untreated	5% ± 1
50 mM AAPH	23% ± 3^***^
0.01 μg/mL Extract + 50 mM AAPH	21% ± 0.1^ns^
50 mM AAPH + 0.01 μg/mL Extract	9% ± 0.5^ns^

### 2.3 Determination of released lactate dehydrogenase (LDH) amount

**FIGURE 3:**
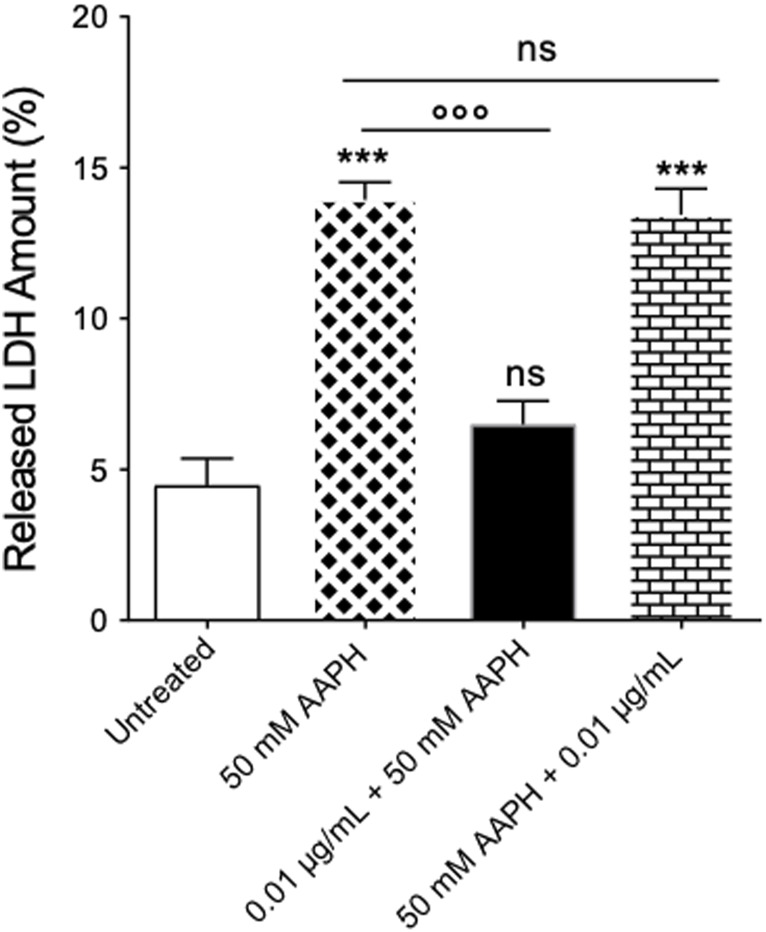
Determination of released LDH amount. Released LDH amounts were detected in RBCs left untreated or incubated with 50 mM AAPH (1 h at 37°C) with or without pre- and post-exposure to the anthocyanin-enriched fraction (0.01 μg/mL) for 1 h at 37°C. ns, not statistically significant *versus* control (untreated) cells and 50 mM AAPH; ***p < 0.001 *versus* untreated; *p* < 0.001 *versus* 50 mM AAPH, one-way ANOVA followed by Tukey’s test (n = 3).

As expected, in RBCs treated with 50 mM AAPH (1 h at 37 °C), a moderate (about 1.8-fold) increase of released LDH was observed compared to cells left untreated (Figure 3). However, pre-exposure of cells to 0.01 μg/mL anthocyanin-enriched fraction (1 h at 37 °C) significantly decreased the amount of LDH released. On the contrary, in RBCs first exposed to 50 mM AAPH (1 h at 37°C) and then incubated with the anthocyanin-enriched fraction (0.01 μg/mL for 1 h at 37°C), the LDH amount released was not different compared to that measured in RBCs treated with 50 mM AAPH. The anthocyanin-enriched fraction alone did not affect the released LDH amount (data not shown).

### 2.4 Measurements of the mean corpuscular volume (MCV)

**FIGURE 4:**
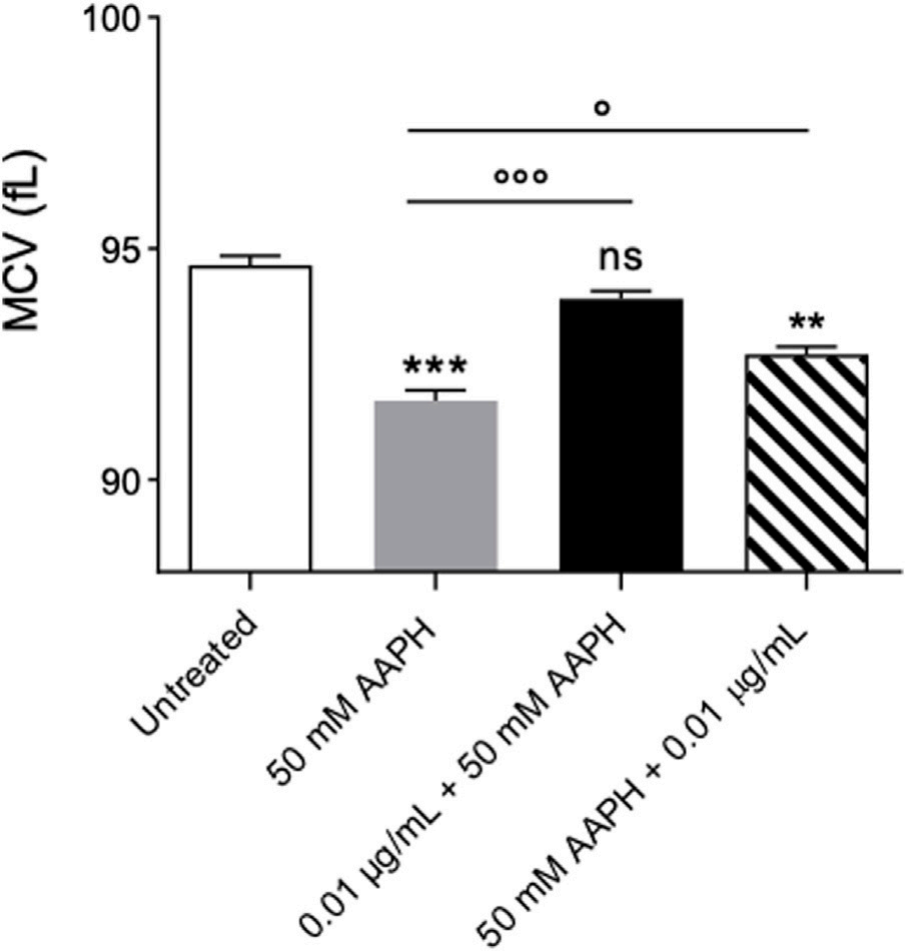
Measurement of Changes in the Mean Corpuscular Volume (MCV). RBCs were left untreated or treated with 50 mM AAPH (1 h at 37°C) with or without pre- and post-exposure to 0.01 μg/mL anthocyanin-enriched fraction for 1 h at 37°C.ns, not statistically significant *versus* untreated; **p < 0.01 and ***p < 0.001 *versus* untreated; *p* < 0.05 and *p* < 0.001 *versus* 50 mM AAPH, one-way ANOVA followed by Bonferroni’s multiple comparison post-hoc test (n = 15).

Figure 4 reports MCV values measured in in left untreated RBCs or treated with 50 mM AAPH (1 h, at 37°C) with or without pre- or post-treatment with anthocyanin-enriched fraction (0.01 μg/mL) for 1 h at 37°C. This parameter is a surrogate measure of the average cellular size. In human RBCs treated with 50 mM AAPH, MCV was significantly lower than those measured in untreated cells. On the contrary, the MCV values in RBCs pre-treated with anthocyanin-enriched fraction were statistically higher than those measured in 50 mM AAPH-treated cells. Instead, the post-treatment with anthocyanin extract was ineffective. Anthocyanin-enriched fraction alone did not affect such parameter.

### 2.5 Oxidative stress assessment

#### 2.5.1 Evaluation of intracellular ROS levels

**FIGURE 5:**
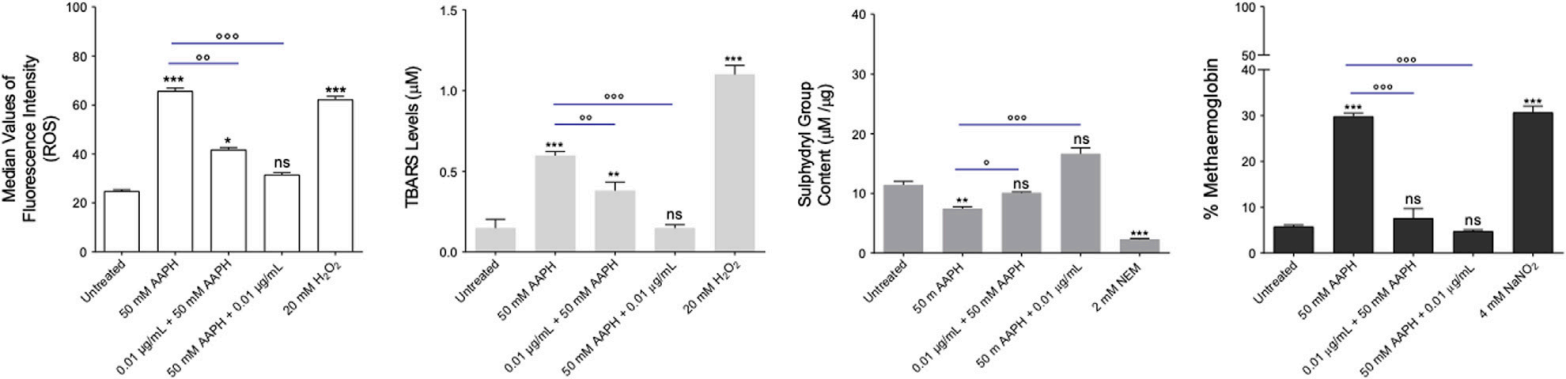
Evaluation of oxidative stress. **(A)** Detection of ROS levels. ROS levels were detected in RBCs left untreated or treated with 50 mM AAPH for 1 h at 37°C with or without pre- or post-exposure to the anthocyanin extract (0.01 μg/mL) for 1 h at 37°C. 20 mM H_2_O_2_ (1 h at 37°C) was used as the positive control. ns, not statistically significant *versus* control (untreated); ***p < 0.001 *versus* control, *p* < 0.01 and *p* < 0.001 *versus* 50 mM AAPH, one-way ANOVA followed by Bonferroni’s post-hoc test (n = 15). **(B)** Estimation of TBARS levels. TBARS levels (µM) were detected in RBCs left untreated or treated with 50 mM AAPH for 1 h at 37°C with or without pre- or post-exposure to the anthocyanin extract (0.01 μg/mL) for 1 h at 37°C. 20 mM H_2_O_2_ (1 h at 37°C) was used as the positive control. ns, not statistically significant *versus* control; **p < 0.01 and ***p < 0.001 *versus* control; *p* < 0.01 and *p* < 0.001 *versus* 50 mM AAPH, one-way ANOVA followed by Bonferroni’s post-hoc test (n = 15). **(C)** Evaluation of sulfhydryl group content. Sulfhydryl group content (µM TNB/µg protein) was detected in RBCs left untreated and in RBCs treated with 50 mM AAPH for 1 h at 37°C with or without pre-or post-exposure to the anthocyanin extract (0.01 μg/mL) for 1 h at 37°C. NEM (2 mM for 1 h at 37°C) was used as a positive control. ns, not statistically significant *versus* control; ***p < 0.001 *versus* control, *p* < 0.01 and *p* < 0.001 *versus* 50 mM APPH, one-way ANOVA followed by Bonferroni’s post-hoc test (n = 15). **(D)** Determination of MetHb (%) levels. MetHb levels were detected in RBCs left untreated or incubated with 50 mM AAPH (1 h, 37°C) with or without pre- or post-exposure to the anthocyanin extract (0.01 μg/mL) for 1 h at 37°C. NaNO_2_ (4 mM for 1 h at 37°C) was used as the positive control. ns, not statistically significant; ***p < 0.001 *versus* control; *p* < 0.001 *versus* 50 mM AAPH, one way ANOVA followed by Bonferroni’s post-hoc test (n = 15).

The measurement of ROS levels was performed by flow cytometry in RBCs that were left untreated or, alternatively, exposed to 50 mM AAPH for 1 h at 37°C with or without pre- or post-exposure to the anthocyanin extract (0.01 μg/mL) for 1 h at 37°C. Figure 5A displays the intracellular ROS levels. Blood samples incubated with AAPH showed a significant increase of intracellular ROS levels compared to untreated samples. In samples exposed to the anthocyanin extract before or after exposure to AAPH, ROS levels were significantly reduced. Notably, in samples first treated with AAPH and successively exposed to the anthocyanin extract, ROS levels did not differ from those of control RBCs. As expected, in RBCs exposed to 20 mM H_2_O_2_ for 1 h at 25°C, the intracellular ROS content was significantly higher than that of control RBCs, whereas the anthocyanin extract (0.01 μg/mL) alone did not affect the production of intracellular ROS (data not shown).

#### 2.5.2 Measurement of thiobarbituric-acid-reactive substances (TBARS) levels

Figure 5B shows the TBARS levels in RBCs left untreated or, alternatively, treated with 50 mM AAPH for 1 h at 37°C with or without pre- or post-exposure to the anthocyanin extract (0.01 μg/mL) for 1 h at 37°C. As expected, TBARS levels of RBCs treated with 20 mM H_2_O_2_ for 1 h were significantly higher than those of untreated RBCs. In parallel, in RBCs pre-incubated with the anthocyanin extract and then treated with AAPH, TBARS production, although significantly reduced compared to RBCs treated with AAPH alone, remained significantly elevated compared to control values. Conversely, in RBCs first incubated with AAPH and then exposed to the anthocyanin extract, TBARS levels were not significantly different from control values. Of note, anthocyanin extract alone did not affect TBARS levels (data not shown).

#### 2.5.3 Measurement of total sulfhydryl group content

Figure 5C displays the total content of sulfhydryl groups in RBCs left untreated or treated with either the oxidizing compound NEM (2 mM for 1 h at 25°C) as the positive control or 50 mM AAPH for 1 h at 37 °C with or without pre- or post-treatment with the anthocyanin extract (0.01 μg/mL) for 1 h at 37°C. NEM incubation led to a significant reduction in sulfhydryl group content compared to control values. Sulfhydryl groups were also significantly reduced in AAPH-treated RBCs. Pre- and post-treatment with the anthocyanin extract completely restored the total levels of sulfhydryl groups. Noteworthy, the anthocyanin extract alone did not induce oxidation of sulfhydryl groups (data not shown).

#### 2.5.4 Evaluation of methemoglobin (MetHb) levels

Figure 5D displays the MetHb levels measured in RBCs left untreated or treated with 50 mM AAPH for 1 h at 37°C with or without pre- or post-treatment with the anthocyanin extract (0.01 μg/mL) for 1 h at 37°C, or alternatively, exposed to the well-known MetHb-forming compound NaNO_2_ (4 mM for 1 h at 25°C). Methemoglobin levels measured after treatment with NaNO_2_ were significantly higher than those detected in untreated RBCs. Alongside, MetHb levels measured after exposure to AAPH were significantly higher than those measured in untreated cells, while both pre- and post-treatment with the anthocyanin extract significantly reduced the production of MetHb levels. The anthocyanin extract alone did not affect MetHb levels.

### 2.6 Detection of protein expression by western blotting analysis

#### 2.6.1 Detection of band 3 protein levels

**FIGURE 6:**
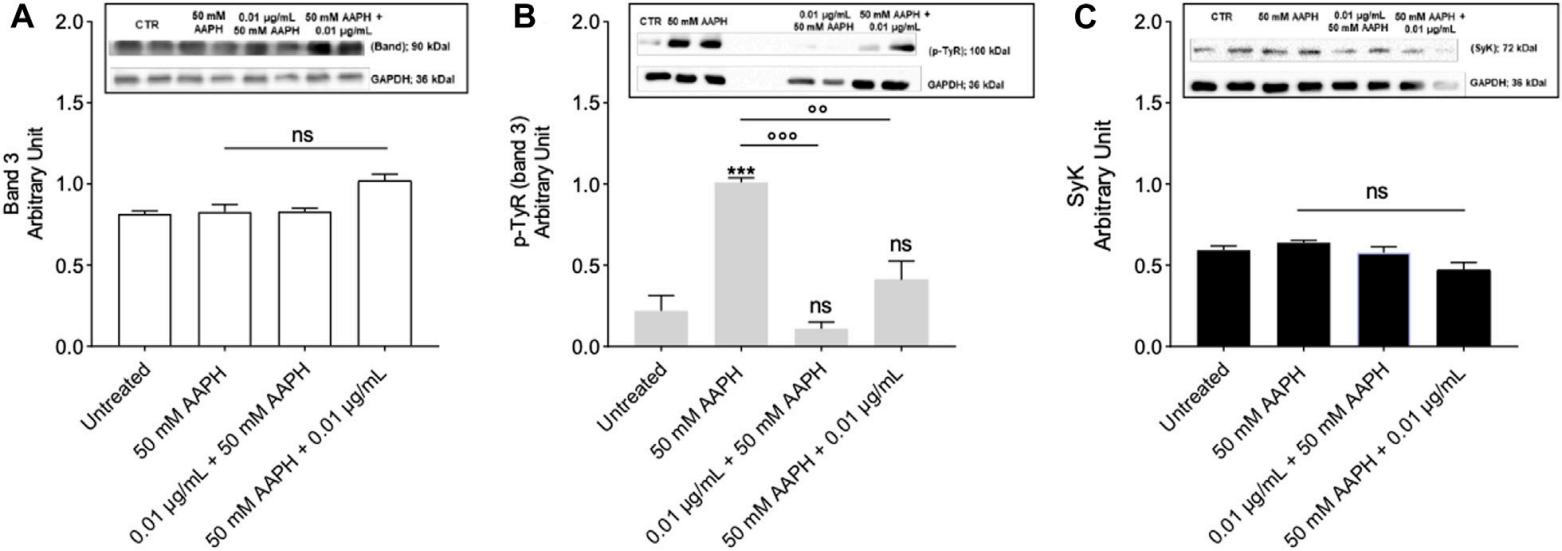
Measurement of Protein Levels by Western blot Analysis. **(A)** Band 3 levels in human RBCs left untreated or exposed to 50 mM AAPH for 1 h at 37°C with or without exposure to 0.01 μg/mL anthocyanin-enriched fraction for 1 h at 37°C; ns not statistically significant *versus* control (untreated), one-way ANOVA followed by Bonferroni’s multiple comparison post-hoc test (n = 3). **(B)** p-Tyr (tyrosine) levels in RBCs left untreated or exposed to 50 mM AAPH for 1 h at 37°C with or without exposure to 0.01 μg/mL anthocyanin-enriched fraction for 1 h at 37°C. ns, not statistically significant; ***p < 0.001 *versus* untreated; *p* < 0.01 and *p* < 0.001 *versus* 50 mM APPH, one-way ANOVA followed by Bonferroni’s multiple comparison post-hoc test (n = 3). **(C)** Syk levels in RBCs left untreated or exposed to 50 mM AAPH for 1 h at 37°C with or without exposure to 0.01 μg/mL anthocyanin-enriched fraction for 1 h at 37°C; ns not statistically significant *versus* control, one way ANOVA followed by Bonferroni’s post-hoc test (n = 3).

Figure 6A shows the levels of band 3 in human RBCs treated with 50 mM AAPH for 1 h at 37°C with or without pre-exposure to 0.01 μg/mL anthocyanin extract for 1 h at 37°C. In all conditions tested, band 3 levels were not significantly different with respect to those determined in untreated RBCs.

#### 2.6.2 Detection of band 3 tyrosine phosphorylation and SyK kinase levels

Figure 6B reports both tyrosine phosphorylation (p-Tyr) levels of band 3 and SyK kinase levels in human RBCs incubated with 50 mM AAPH for 1 h at 37°C with or without pre-incubation with the anthocyanin-enriched fraction (0.01 μg/mL) for 1 h at 37°C. Exposure of RBCs to AAPH caused an intense band 3 phosphorylation (Figure 6B). Importantly, treatment with the anthocyanin-enriched fraction before or after exposure to 50 mM AAPH prevented the increase of tyrosine phosphorylation of band 3 (Figure 6B). Anthocyanin-enriched fraction alone did not significantly affect tyrosine phosphorylation levels (data not shown). In parallel, Syk kinase, which is responsible for the phosphorylation of band 3, was detected (Figure 6C). No Syk kinase expression changes were detected in human RBCs treated with 50 mM AAPH, or alternatively, exposed to the anthocyanin-enriched fraction before or after exposure to AAPH. Also, anthocyanin-enriched fraction alone did not significantly modify Syk kinase expression (data not shown).

### 2.7 Band 3 exposition level determination by flow cytometry analysis

**FIGURE 7:**
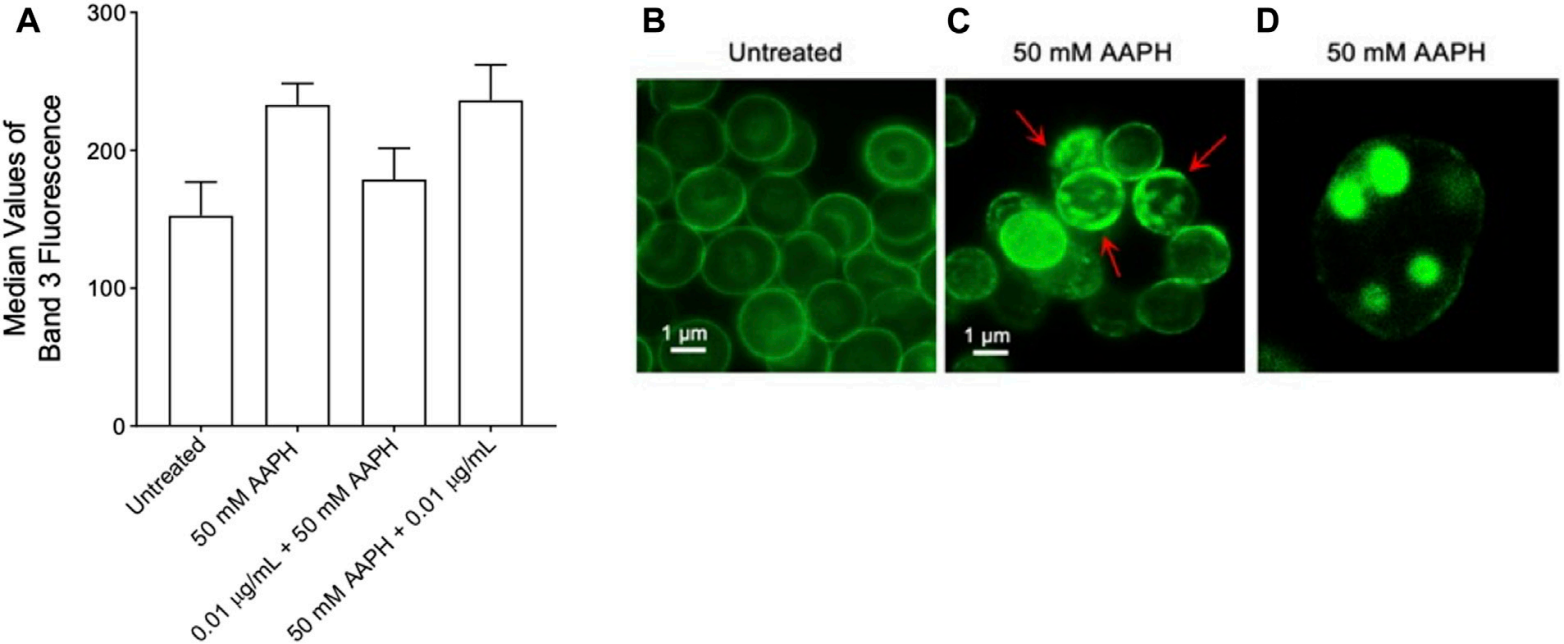
Flow cytometry immunofluorescence of band 3 levels. Red blood cells were left untreated or treated with 50 mM AAPH for 1 h at 37°C, with or without pre- and post-exposure to 0.01 μg/mL anthocyanin-enriched fraction for 1 h at 37°C. **(A)** Histograms reporting median values of fluorescence intensity. **(B-D)** Flow cytometry immunofluorescence representative micrographs showing band 3 distribution in untreated RBCs and RBCs treated with 50 mM AAPH. Samples were observed with a ×100 objective. **(B)** Significant morphological changes in 50 mM AAPH-treated RBCs were reported (red arrows). **(C)** Hemichromes formation is reported (7,000x magnification). ns, not statistically significant *versus* untreated, ANOVA followed by Bonferroni’s multiple comparison post-test (*n* = 10).

**FIGURE 8:**
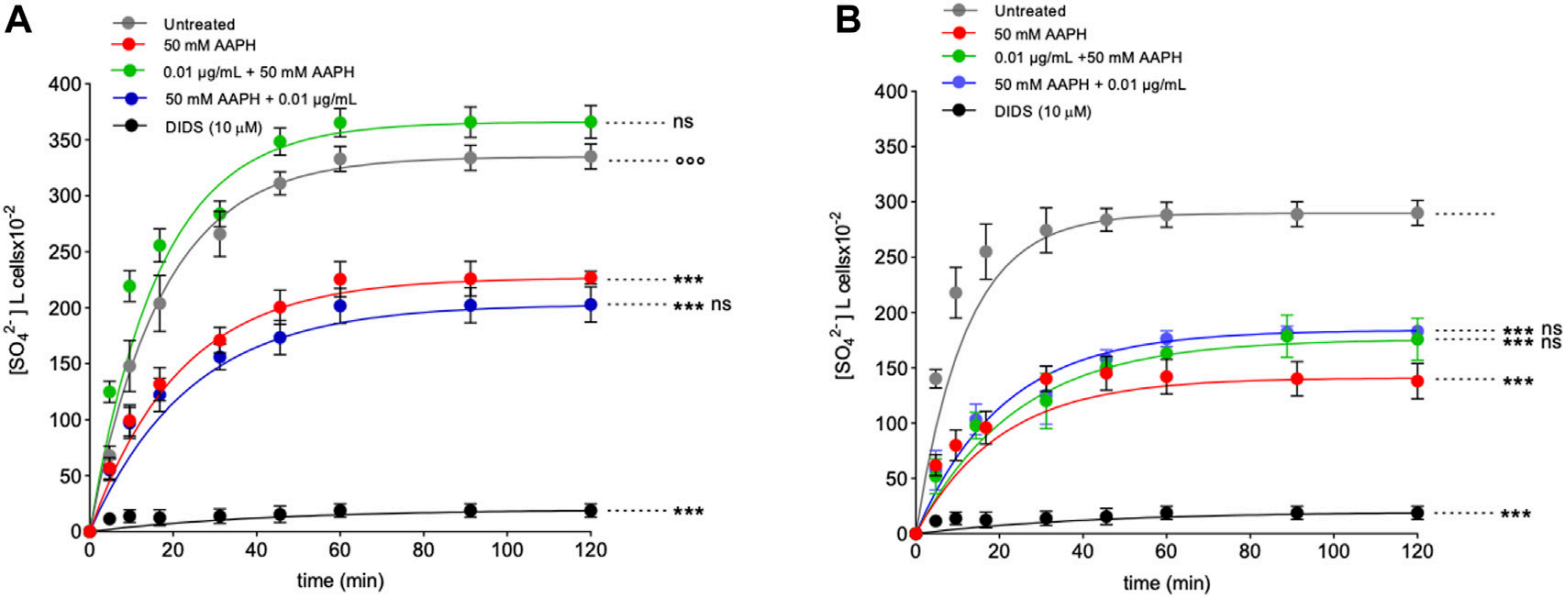
Time course of SO_4_^2^⁻ uptake. **(A)** Intact RBCs were left untreated or treated with 50 mM AAPH (1 h, at 37°C) with or without pre- and post-exposure to 0.01 μg/mL anthocyanin-enriched fraction for 1 h at 37°C, or alternatively, exposed to 10 µM DIDS. ns, not statistically significant *versus* untreated and 50 mM AAPH; ***p < 0.001 *versus* control; *p* < 0.001 *versus* 50 mM AAPH, one way ANOVA followed by Bonferroni’s post-hoc test. **(B)** Resealed ghosts were left untreated or treated with 50 mM AAPH (1 h at 37°C), or alternatively, exposed to 10 µM DIDS. ***p < 0.001 *versus* untreated; one-way ANOVA followed by Bonferroni’s post-hoc test.

Expression levels of band 3 (median values of fluorescence obtained by flow cytometry) observed in human RBCs exposed to the anthocyanin-enriched fraction or to AAPH were comparable to those reported in untreated RBCs (Figure 7A). Although data were not statistically significant, an increased exposition of band 3 was detected in human RBCs treated with 50 mM AAPH for 1 h at 37°C with respect to untreated cells. Representative immunofluorescence images of band 3 distribution in untreated cells (left panel) and in 50 mM AAPH-treated RBCs (middle and right panels) are shown, respectively. Results demonstrated that band 3 was mainly clustered (red arrows) in acanthocytes after 50 mM AAPH treatment with respect to untreated RBCs (Figure 8B). Moreover, band 3 exposition was not altered by the anthocyanin-enriched fraction given alone (data not shown).

### 2.8 Measurement of SO_4_^2−^ uptake through band 3 in intact RBCs

Figure 8 shows the SO_4_^2−^ uptake as a function of time in RBCs untreated and in RBCs treated with 50 mM AAPH for 1 h at 37°C with or without pre- or post-treatment with 0.01 μg/mL anthocyanin-enriched fraction for 1 h at 37°C. In untreated samples, SO_4_^2^⁻ uptake gradually increased and reached equilibrium within 45 min (0.057 ± 0.001 min^−1^). Blood samples treated with the anthocyanin-enriched fraction alone showed a rate constant of SO_4_^2−^ uptake not significantly different with respect to control (data not shown). On the contrary, the rate constant (0.046 ± 0.001 min^−1^) in RBCs treated with AAPH was significantly decreased with respect to control (***p < 0.001). Notably, in RBCs pre-incubated with the anthocyanin-enriched fraction and then treated with 50 mM AAPH, the rate constant (0.060 ± 0.001 min^−1^) was significantly higher (*p* < 0.001) than that of RBCs treated with AAPH alone and was not significantly different with respect to control (Table 3). In contrast, in RBCs first incubated with AAPH and then treated with the anthocyanin-enriched fraction, the rate constant (0.041 ± 0.001 min^−1^) was not significantly different with respect to that of RBCs treated with AAPH alone (Table 3). SO_4_^2^⁻ uptake via band 3 was almost totally blocked by DIDS (10 µM) applied at the beginning of incubation in SO_4_^2^⁻ medium (0.04 ± 0.001 min^−1^, ***p < 0.001, Table 3). In addition, the SO_4_^2^⁻ amount internalized by AAPH-treated RBCs after 45 min of incubation in SO_4_^2^⁻ medium was significantly lower than untreated (Table 3). Conversely, the SO_4_^2^⁻ amount internalized by RBCs pre-incubated with the anthocyanin-enriched fraction and then exposed to AAPH was not significantly different compared to that measured in untreated RBCs (Table 3). In DIDS-treated samples, the SO_4_^2^⁻ amount internalized was significantly lower than that of both untreated and treated RBCs (***p < 0.001, Table 3).

**TABLE 3:** Rate constant of SO_4_^2^⁻ uptake and amount of SO_4_^2^⁻ trapped in human RBCs untreated and RBCs treated as indicated. Results are presented as means ± SEM. from separate experiments (n), where ns, not statistically significant *versus* untreated and/or 50 mM AAPH; ***p 0.001 *versus* untreated (intact RBCs, or alternatively, in resealed ghosts); *p* < 0.001 *versus* 50 mM AAPH, one-way ANOVA followed by Bonferroni’s multiple comparison post-hoc test.

Experimental conditions	Rate constant (min^-1^)	TIME (min)	n	SO_4_^2-^ amount trapped after 45 min of incubation IN SO_4_^2-^ medium [SO_4_^2-^] l cells x10^−2^
INTACT RBCS				
Untreated	0.057 ± 0.001	17.16	10	311 ± 8.95
50 mM AAPH	0.046 ± 0.001^***^	21.44	10	200 ± 10.25^***^
0.01 μg/mL Extract + 50 mM AAPH	0.060 ± 0.003^ns^	16.43	10	348 ± 18.65 ^ns^
50 mM AAPH + 0.01 μg/mL Extract	0.041 ± 0.002^ns,***^	23.8	10	173 ± 11.37 ^ns,***^
10 µM DIDS	0.024 ± 0.001^***^	41.66	10	15.5 ± 0.37^***^
RESEALED GHOSTS				
Untreated	0.049 ± 0.001^***^	12.01	10	284 ± 0.35
50 mM AAPH	0.082 ± 0.002^***^	20.40	10	142.3 ± 0.50^***^
0.01 μg/mL Extract + 50 mM AAPH	0.048 ± 0.002^***, ns^	20.62	10	158.05 ± 0.42^***,ns^
50 mM AAPH + 0.01 μg/mL Extract	0.041 ± 0.002^***, ns^	23.87	10	150.05 ± 0.32^***,ns^
10 µM DIDS	0.023 ± 0.001^***^	43.47	10	14.3 ± 0.29^***^

#### 2.8.1 Measurement of SO_4_^2−^ uptake through band 3 in resealed ghosts

Figure 8B reports the time course of SO_4_^2^⁻ uptake in resealed ghosts. The rate constant of SO_4_^2^⁻ uptake (0.082 ± 0.002 min^−1^, Table 3) in RBCs treated with 50 mM AAPH (1 h, at 37°C) was significantly decreased with respect to untreated RBCs (0.049 ± 0.001, ***p < 0.001, Table 3). SO_4_^2^⁻ uptake via band 3 was almost totally blocked by DIDS applied at the beginning of incubation in SO_4_^2^⁻ medium (0.024 ± 0.001 min^−1^, ***p < 0.001, Table 3). In addition, the SO_4_^2^⁻ amount internalized by AAPH-treated RBCs after 45 min of incubation in SO_4_^2^⁻ medium was significantly lower than the control (Table 3). In DIDS-treated samples, the SO_4_^2^⁻ amount internalized was significantly lower than both untreated and treated RBCs (***p < 0.001, Table 3). In RBCs pre-incubated with the anthocyanin extract and then treated with 50 mM AAPH, the rate constant (0.048 ± 0.001 min^−1^) was not different than that of RBCs treated with 50 mM AAPH alone and was significantly different with respect to control (Table 3). In parallel, in RBCs first incubated with 50 mM AAPH and then treated with the anthocyanin-enriched fraction, the rate constant (0.041 ± 0.001 min^−1^) was not significantly different with respect to that of human RBCs treated with 50 mM AAPH alone (Table 3). SO_4_^2^⁻ uptake via band 3 was almost totally blocked by DIDS applied at the beginning of incubation in SO_4_^2^⁻ medium (0.023 ± 0.001 min^−1^, ***p < 0.001, Table 3). In addition, the SO_4_^2^⁻ amount internalized by AAPH-treated RBCs after 45 min of incubation in SO_4_^2^⁻ medium was significantly lower than untreated cells (Table 3). Also, the SO_4_^2^⁻ amount internalized by RBCs pre- and post-incubated with the anthocyanin-enriched fraction and exposed to 50 mM AAPH was significantly different compared to that measured in untreated RBCs (Table 3). In DIDS-treated samples, the SO_4_^2^⁻ amount internalized was significantly lower than that of both untreated and treated RBCs (***p < 0.001, Table 3).

### 2.9 Assessment of the Endogenous Antioxidant Activity

#### 2.9.1 Determination of reduced glutathione (GSH) levels

**FIGURE 9:**
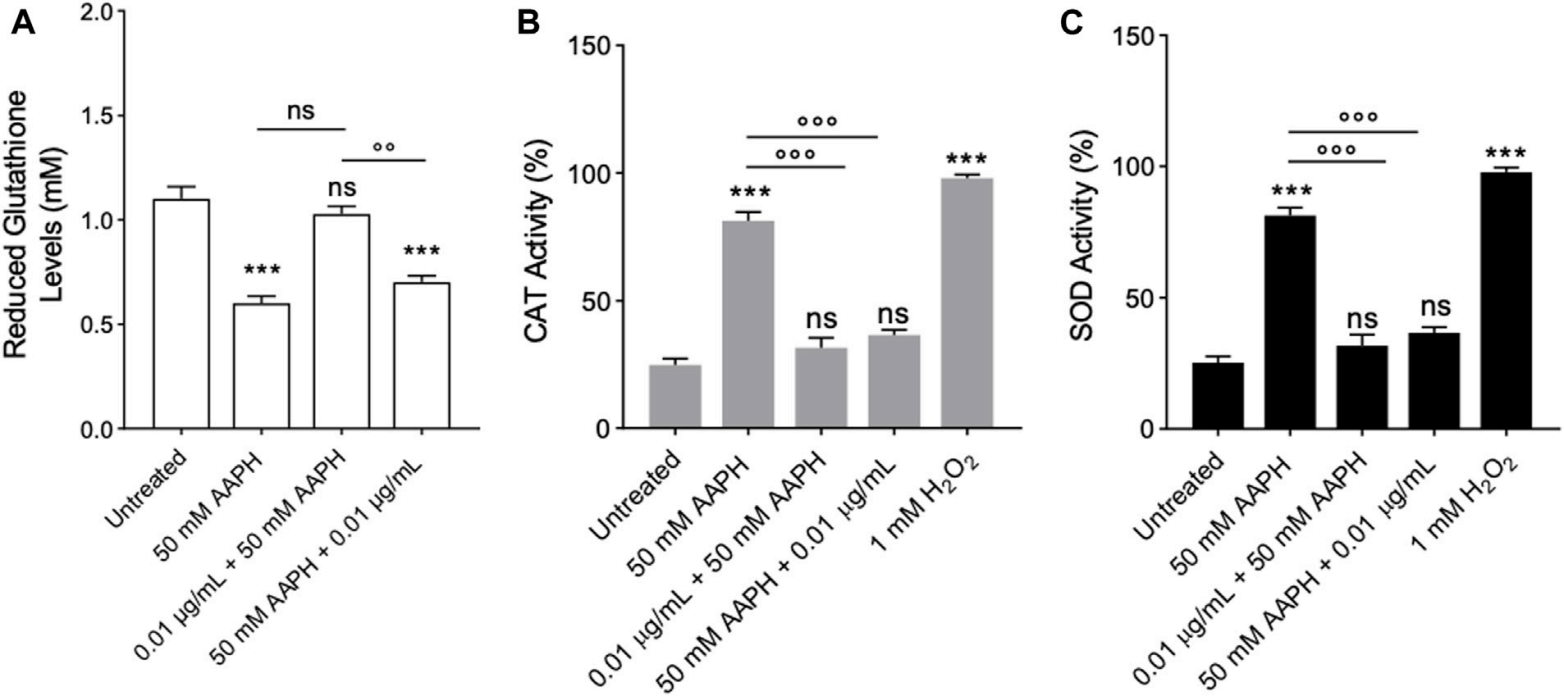
Evaluation of Endogenous Antioxidant Activity. Red blood cells were treated with 50 mM AAPH for 1 h at 37°C with or without pre- and post-treatment with the anthocyanin-enriched fraction (1 h at 37°C)**. (A)** ns, not statistically significant *versus* untreated RBCs and 50 mM AAPH; ***p < 0.001 *versus* untreated; *p* < 0.01 *versus* pre-treatment, one-way ANOVA followed by Tukey’s test. (n = 8). **(B)** CAT activity and **(C)** SOD activity. ns, not significant *versus* untreated RBCs; ***p < 0.001 *versus* untreated RBCs; *p* < 0.001 *versus* 50 mM AAPH, one-way ANOVA followed by Bonferroni’s multiple comparison post-hoc test (n = 8).

Figure 9A reports the GSH levels measured in human RBCs treated with 50 mM AAPH for 1 h at 37°C with or without pre- and post-treatment with the enriched anthocyanin fraction (0.01 μg/mL for 1 h at 37°C). The reduced GSH levels measured after incubation with AAPH were significantly lower (∼45%) than those detected in untreated RBCs. Nevertheless, pre-incubation with the anthocyanin-enriched fraction completely restored the GSH content. On the contrary, in human RBCs first exposed to 50 mM AAPH (1 h at 37°C) and then treated with the anthocyanin-enriched fraction (0.01 μg/mL for 1 h at 37°C), the GSH content was not different compared to that measured in RBCs treated with 50 mM AAPH. The anthocyanin-enriched fraction alone did not significantly modify the GSH levels (data not shown).

#### 2.9.2 Catalase (CAT) activity assay

**FIGURE 10:**
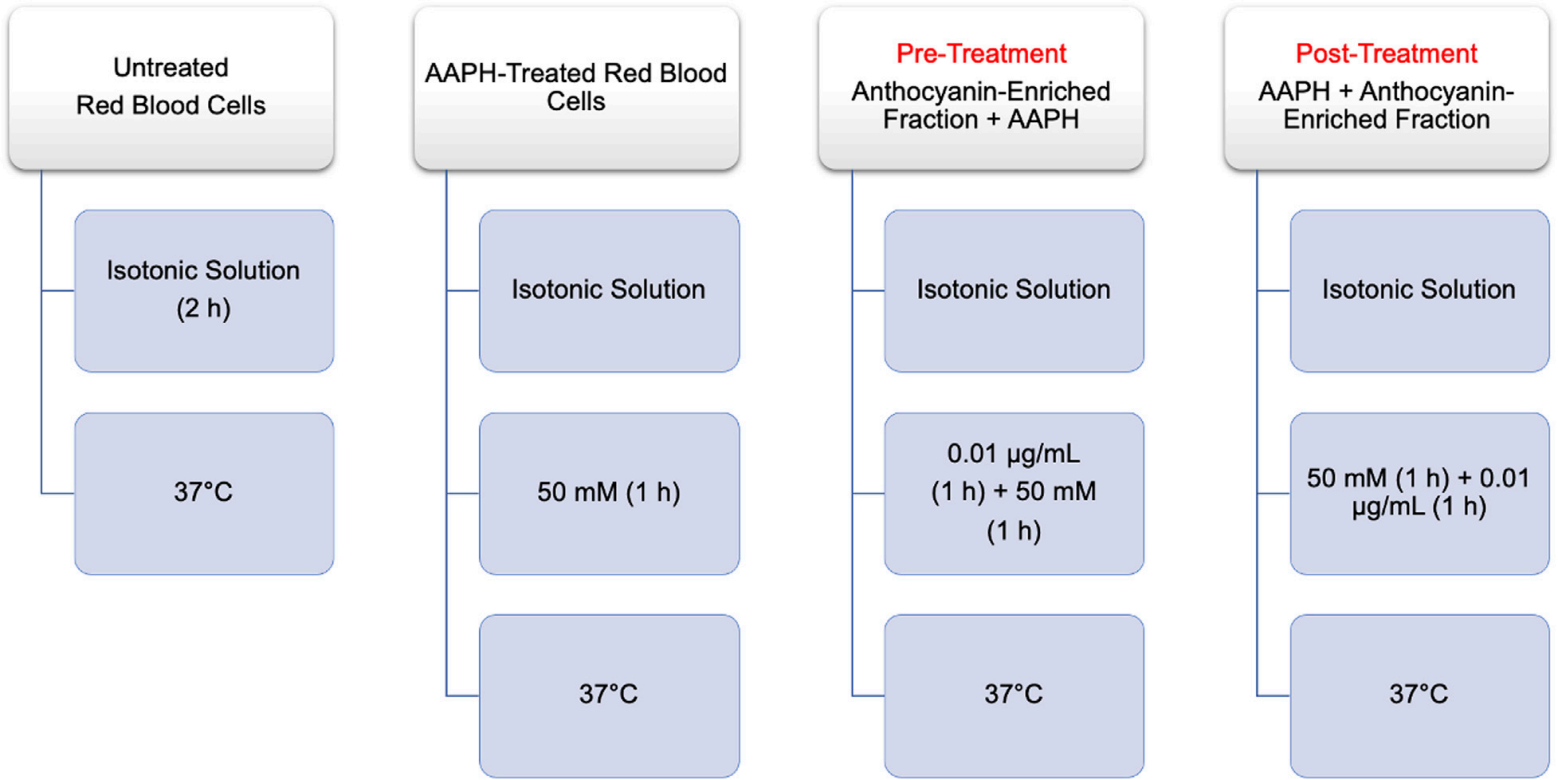
Time course of experimental procedures. AAPH: 2,2′-Azobis (2-methylpropionamidine) dihydrochloride.

Catalase was assayed in human RBCs untreated or treated with 50 mM AAPH for 1 h at 37°C with or without pre- or post-treatment with the anthocyanin-enriched fraction (0.01 μg/mL) for 1 h at 37°C. The treatment with AAPH provoked an increased CAT activity compared to cells left untreated, which was consistent with an elevated oxidative stress (Figure 10A). Unlike, the pre- and post-incubation with anthocyanin-enriched fraction (0.01 μg/mL for 1 h at 37 °C) reduced the CAT activity to control values (Figure 9B). Exposure to 1 mM H_2_O_2_ for 30 min at 25°C has been considered as a positive control. As expected, CAT activity in human RBCs treated with H_2_O_2_ was significantly higher than that of control RBCs, whereas anthocyanin-enriched fraction alone did not significantly modify CAT activity (data not shown).

#### 2.9.3 Superoxide dismutase (SOD) activity assay

In Figure 9C, the SOD activity was measured in human RBCs untreated or treated with 50 mM AAPH for 1 h at 37°C with or without pre- or post-treatment with the anthocyanin-enriched fraction (0.01 μg/mL) for 1 h at 37 °C. In AAPH-treated RBCs, SOD activity was found significantly increased compared to the cells left untreated. On the contrary, both pre- and post-incubation with the anthocyanin extract resulted in a significant inhibition of SOD activity with respect to AAPH-treated cells. As expected, SOD activity in RBCs treated with 1 mM H2O2 (30 min at 25 °C) was significantly higher than that of control RBCs. Anthocyanin-enriched fraction did not significantly modify SOD activity when given alone (data not shown).

## 3 Discussion

Red blood cells are the most numerous cells of the human blood. Their discoid donut-like shape is essential for their physiological functions as it enhances cellular flexibility and favours a high cellular surface area-to-volume ratio, thus allowing efficient gas (O_2_ and CO_2_, respectively) exchange from the lungs to the tissues and *vice versa* (Sicinska, 2018). However, these cells are constantly exposed to stressors during their 120-day lifespan, resulting in biochemical and structural modifications. These alterations could impair the ability of the RBCs to transport oxygen and eventually trigger their removal from the blood circulation (Kuhn et al., 2017). The processes triggering RBC removal have been partly investigated. As many of these processes involve the increase of oxidative stress levels, the present investigation aimed to explore the cellular and molecular mechanisms underlying oxidative stress in human AAPH-stimulated RBCs. To achieve this aim, the relationship between RBC morphology and functional activity has been explored.

Plant-based antioxidant compounds could help reduce the effect of increased ROS levels and the resultant oxidative stress and can boost the endogenous antioxidant system against oxidizing molecules, thus playing a crucial role in the prevention of oxidative stress-related pathological states (Ugusman et al., 2023). In this context, the potential protective activity of an anthocyanin-enriched fraction extracted from *Callistemon citrinus* flowers (Table 1) for counteracting oxidative stress events was also studied. An interesting issue concerning the benefits of phytochemicals in human health is their combined administration. Combinations of several phytochemicals can cause a change in both final biological effects and bioavailability of each component. These combinations can improve or reduce the benefits conferred by individual bioactive compounds, as well as may induce facilitation/competition for cellular absorption and transport (Wang et al., 2011). Synergistic interactions between antioxidants can be explained by the theory of antioxidant regeneration: one antioxidant protects another antioxidant from oxidative degradation or isomerization by its own oxidation. In this context, Bendokas and collaborators stated that a complex mixture of anthocyanin metabolites in the plasma rather than a single type of anthocyanins may cause beneficial effects in humans (Bendokas et al., 2020).

The possible beneficial effect has been assayed by applying the extract either before treatment of RBCs with the established pro-oxidant AAPH (pre-treatment) or after treatment with AAPH (post-treatment) Although multiple investigations reported the beneficial properties of extracts of plant origin, the anthocyanin effects on oxidative stress events in human RBCs have not yet been fully investigated.

The susceptibility of RBCs to AAPH exposure was investigated in terms of morphological changes by scanning electron microscopy (SEM). The images (Figure 2) revealed significant changes in the cellular shape, as the canonical biconcave shape was lost in a notable number of cells, which displayed surface blebs known as acanthocytes. However, post-treatment with the anthocyanin-enriched fraction attenuated the morphological modifications (Figure 2), with a significant reduction of the acanthocyte percentage. The morphology of circulating RBCs has a fundamental influence on the rheological properties of the blood (Gyawali et al., 2012; Gyawali et al., 2015). The cell integrity, one of the major determinants of blood rheological properties, implies that human RBCs are capable of reversible deformation in the different conditions of shear in the bloodstream. In this context, LDH release is commonly considered as a marker of loss of cell membrane integrity in response to elevated oxidant stress levels. This enzyme is normally compartmentalized within the RBCs, but its activity is significantly increased in the extra-cellular side as a result of cellular damage (Franca et al., 2014; Morabito et al., 2014). To better explore this characteristic, the amount of released LDH was also quantified. As expected, in human RBCs treated with 50 mM AAPH a moderate increase in released LDH was detected (Figure 3). However, the pre-exposure to the anthocyanin-enriched fraction significantly decreased the released LDH amount, which returned to control values. Conversely, in RBCs first exposed to 50 mM AAPH and then incubated with the anthocyanin-enriched fraction, the amount of released LDH was not different compared to that measured in 50 mM AAPH-treated RBCs, but was significantly higher than that of untreated RBCs. These results indicate that anthocyanins can prevent but not reverse an oxidative stress-induced loss of cell membrane integrity. Red blood cells membrane mechanical properties can be remodeled by oxidative stress, thus resulting in a reduction of deformability, or alternatively, in altered permeability of the phospholipid bilayer, which, in turn, limits plasma membrane ability to counter osmotic variations (Spinelli et al., 2023). Among the main determinants of RBC deformability (Maruyama et al., 2022), the mean corpuscular volume (MCV, as a measure of cellular size) was considered. Our results demonstrated that pre-treatment with the anthocyanin-enriched fraction reverted the cell size reduction provoked by exposure to AAPH (Figure 4). The reduced membrane surface area following oxidative stress was confirmed by both reduced cell size (MCV) and the presence of acanthocytes (Figure 4).

It is interesting to point out that oxidative stress events, as well as oxidative stress-related diseases (Remigante et al., 2021b; Zuccolini et al., 2022), are associated with RBC shrinkage (Rinalducci et al., 2011). In 𝛽-thalassemic RBCs, a pro-oxidant environment favours the abnormal band 3 clusterization, inhibition of the Ca-ATPase pump, and activation of Ca-permeable unselective cation channels (Crupi et al., 2010; Voskou et al., 2015). The consequent increase in intracellular Ca^2+^ activates the K^+^/Cl^−^ co-transporter (KCC), which causes the leakage of K^+^ from the cell and results in cellular shrinkage and impaired deformability. An increase in intracellular calcium also activates calpain and some caspases that can degrade and/or crosslink cytoskeletal proteins and lead to eryptosis (De Franceschi et al., 2013). Eryptosis mimics the programmed cell death of nucleated cells (apoptosis). This phenomenon is characterized by a gradual increase in membrane phospholipid asymmetry and ATP consumption, which results in the externalization of phosphatidylserine to the outer leaflet of the plasma membrane (Yasin et al., 2003; Bosman et al., 2011). The externalization of phosphatidylserine on the surface of eryptotic cells can have two pathophysiological consequences: on one hand, it starts the RBC phagocytosis; on the other hand, it favours RBC adherence to vascular endothelial cells, which express phosphatidylserine receptors. Exposure to AAPH did not induce the translocation of phosphatidylserine at the outer plasma membrane leaflet (Supplementary Figure S2), thus demonstrating that, in this model of acute oxidative stress, human RBCs remain in an early and vital phase of the oxidizing process. Human RBCs may respond to any form of insult by changing their morphology following alterations in the biochemical composition of the plasma membrane. Red blood cells are extremely susceptible to ROS-induced injury because of their high polyunsaturated fatty acid content and their abundance in iron (Fe^2+^)-rich haemoglobin. The latter acts as a catalyst in redox reactions and lipid peroxidation, resulting in TBARS as the final product (Pandey and Rizvi, 2010). Also, RBCs often undergo plasma membrane protein oxidation (Tsamesidis et al., 2020). Therefore, the oxidation of protein sulfhydryl groups is a typical indicator of oxidative damage at the protein level in human RBCs. These phenomena could alter plasma membrane properties and, consequently, cell shape. Since ROS generated during cellular metabolism cause the oxidation of macromolecules, the effects of the anthocyanin extract on the intracellular ROS content have been evaluated. Our results show that both pre- and post-treatment with the anthocyanin-enriched fraction induced a reduction of ROS generation induced by AAPH (Figure 5A). Also, pre- and post-treatment with the anthocyanin-enriched fraction avoided the lipid peroxidation of plasma membranes caused by AAPH (Figure 5B) and protected RBC protein sulfhydryl groups from oxidative injury (Figure 5C). These results demonstrate the antioxidant capacity of anthocyanins. These plant-derived components properly protect both the lipid and protein components of RBC membrane from oxidative injury, and may act as scavengers for neutralizing both reactive species and free radicals. These data are in line with what was previously reported by other authors (Heo and Lee, 2005; Fernandes et al., 2013; Speer et al., 2020; Hu J. et al., 2023; Hu X. et al., 2023).

Band 3 (SLC4A1/AE1) is the dominant integral transmembrane protein in the human RBCs (Mohandas and Gallagher, 2008). This protein plays different important functions, such as a) maintenance of anion homeostasis through the C-terminal domain that carries out Cl-/HCO_3_^−^ exchange across the plasma membrane (Reithmeier et al., 2016), b) maintenance of cell shape because of the binding between the plasma membrane and cytoskeletal structures (Vallese et al., 2022), and c) maintenance of the interaction of some cytosolic proteins with the plasma membrane through the N-terminal domain that extends into the intracellular side. In particular, this region of band 3 competitively binds both haemoglobin and glycolytic enzymes (Issaian et al., 2021). The band 3 function can be investigated via the measurement of the rate constant for sulfate (SO_4_^2−^) uptake (Morabito et al., 2016; Morabito et al., 2017a; Morabito et al., 2017b; Morabito et al., 2018; Remigante et al., 2020; Remigante et al., 2022c; Remigante et al., 2022d; Remigante et al., 2022e), which is slower and more easily measurable than the physiological uptake of Cl^−^ or HCO_3_^−^ (Jennings, 1976; Romano and Passow, 1984; Morabito et al., 2019a; Crupi et al., 2019). This methodological approach is as a valid tool to study the impact of oxidative stress on mature RBCs homeostasis (Morabito et al., 2016; Morabito et al., 2019a; Remigante et al., 2019; Remigante et al., 2022a; Remigante et al., 2023b; Perrone et al., 2023). Based on this background, SO_4_^2−^ uptake through band 3 was measured in mature RBCs after exposure to AAPH with or without pre- and post-treatment with the anthocyanin-enriched fraction. In RBCs incubated with AAPH, the rate constant for SO_4_^2−^ uptake was decreased compared to the untreated cells (Figure 7A; Table 3). The finding that increased oxidative stress caused functional alterations of band 3 activity in mature RBCs has been demonstrated also in other cell-based models of oxidative stress. To name just an example, Morabito and collaborators have demonstrated that not haemolytic concentrations of H_2_O_2_ (300 μM, for 30 min, at 37°C) induced a reduction of band 3 transport efficiency (Morabito et al., 2017b). Such a reduction, which was associated with significant oxidative stress, could be attenuated by a short-time pre-exposure of mature RBCs to low (10 μM, for 10 min at 37°C) H_2_O_2_ concentrations. The pre-exposure induces RBCs to adapt to a mild and transient oxidative stress and allows for an increased endurance to a subsequent stronger oxidant condition. Such an adaptation response, known as pre-conditioning, did not involve the phosphorylation (p-Tyr) pathways of band 3 but is mediated by the increase of CAT activity. In fact, this strategy enabled RBCs to improve the endogenous antioxidant defence performance, in order to provide a better protection against oxidative injury (Morabito et al., 2017b). Although no alterations in band 3 protein expression were reported in RBCs treated with AAPH with or without exposure to the anthocyanin-enriched fraction (Figure 7A), the pre-exposure of RBCs previously treated with AAPH to the anthocyanin-enriched fraction totally restored the rate constant for SO_4_^2−^ uptake (Figure 7A; Table 3). On the contrary, the post-treatment did not enable recovery of the rate constant of SO_4_^2−^ uptake (Figure 7A; Table 3).

Based on data hitherto showed, we can confirm that the anthocyanin extract displays a beneficial effect on the anionic exchange and could play a crucial role in counteracting oxidative stress-related functional modifications in mature RBCs. In elevated oxidative stress conditions, phosphorylation of proteins acts a crucial role in the modulation of the plasma membrane elasticity, resulting in the deformability of mature RBCs. For instance, tyrosine phosphorylation of the band 3 cytoplasmic domain breaks the interaction between band 3 and ankyrin, which connects the cytoskeleton spectrin to the plasma membrane, and induces metabolic changes via the reduction of anion transport (Cilek et al., 2023). The redox regulation of band 3 (p-Tyr) phosphorylation requires the action of two specific enzymes, Lyn, which allows for tyrosine 359 phosphorylation, and Syk, which allows for tyrosine 8 and 21 phosphorylation (Brunati et al., 2000; Bordin et al., 2009; Marchetti et al., 2020). It is well established that post-translation modifications (Costa et al., 2020) can impair cytoskeleton (spectrin)-band 3 binding and provoke modifications in the deformability and cell shape of mature RBCs (Lin and Brown, 2005). In this regard, AAPH-caused oxidative stress provoked a significant increase of band 3 tyrosine phosphorylation. However, both pre- and post-treatment with the anthocyanin-enriched fraction avoided these post-translational modifications (Figure 6B). Overall, no alteration in the Syk kinase expression levels was shown after treatment with AAPH with or without pre- and post-incubation with the anthocyanin-enriched fraction (Figure 6C). The oxidation of the band 3 cytoplasmatic domain is likely induced by the increase of the intracellular ROS production. Elevation of ROS allows for Syk docking to band 3 and suppresses the action of the tyrosine phosphatases (Pantaleo et al., 2016; Pantaleo et al., 2017). It is well known that the binding site for oxidized haemoglobin (MetHb) is also located in the band 3 N-terminal cytoplasmic domain (Mollan et al., 2013). In RBCs, the production of ROS favours the haemoglobin denaturation, resulting in the release of heme iron (Reeder, 2023). This process can be auto-catalytic, thus leading to ever-increasing oxidative stress. To better explore the molecular relationship between band 3 and oxidized haemoglobin, MetHb levels were assayed. Our findings showed that exposure to AAPH increased the levels of MetHb in mature RBCs (Figure 5D). These modifications can initiate a cascade of biochemical and/or structural changes, including the release of micro-particles containing both hemichromes and clustering of band 3 regions (Low et al., 1985; Mannu et al., 1995; Pantaleo et al., 2016). When intracellular oxidants are produced in excess, the balance between antioxidant and pro-oxidant capacity can be modified. Here, band 3 clusters could facilitate the recognition by antibodies directed against aging cells, thus triggering the premature removal of senescent RBCs from circulation before the physiological end of their 120-day life span. Interestingly, both pre- and post-treatment with the anthocyanin-enriched fraction prevented AAPH-induced MetHb production (Figure 5D). As mentioned above, another special feature of RBC oxidation is the band 3 clustering process. To better clarify band 3 distribution in conditions of elevated oxidative stress, the exposition of this protein was investigated by immunofluorescence analysis. Data displayed that, following exposure to AAPH, band 3 re-arranged in surface blebs, thus leading to the formation of clustered band 3 (Figures 8C,D). In particular, Figure 8C shows an aggregation of fluorescent bodies, most probably caused by the association between band 3 and MetHb. These data confirmed that MetHb binding to the C-terminus domain of band 3 induces clustering of band 3, thus leading to hemichromes formation. Yet, despite this, band 3 exposition levels were recovered in RBCs pre- and post-treated the anthocyanin-enriched fraction (Figure 8A).

To confirm the hypothesis that intracellular content could be involved in the AAPH-induced oxidative stress response, we measured band 3 exchange capability in resealed ghosts, which are composed of reconstituted plasma membranes but deprived of intracellular cytoplasmatic content. Resealed ghosts represent a valid tool for investigating band 3 activity as well as its interaction with cytoplasmic proteins (Morabito et al., 2016). In resealed ghosts, the exposure to AAPH induced a reduction of the rate constant of SO_4_^2-^ uptake with respect to untreated ghosts (Figure 7B; Table 3), thus suggesting that the prime target of AAPH seems to be the lipid component of the plasma membrane. The pre- and post-treatment with the anthocyanin-enriched fraction did not restore the anion exchange capability through band 3 (Figure 5B). In these experimental conditions, human RBCs were deprived of intracellular cytoplasmatic content, including methaemoglobin reductase. Normally, this enzyme converts methaemoglobin back to haemoglobin (Kuma, 1981). As demonstrated, haemoglobin is also able to bind the N-terminal domain of band 3 and supports the anion exchange activity. However, the lack of methaemoglobin reductase in AAPH-treated resealed RBCs did not allow for such a conversion, resulting in an altered band 3 function (Figure 7B). The data obtained indicate that the anthocyanin-enriched fraction may protect the enzyme activity, including that of methaemoglobin reductase, in order to reduce excessive oxidative stress levels. Not surprisingly, mature RBCs are equipped with a battery of different antioxidant mechanisms, a combination of both antioxidant enzymes and non-enzymatic compounds, to inactivate the oxidizing species (Moller et al., 2023). Such efficient detoxification system keeps RBCs functional for 120 days in the bloodstream. Antioxidant enzymes possess an excellent free radical scavenging capacity and play crucial roles in mature RBCs (Ulanczyk et al., 2020). Glutathione is the main non-enzymatic endogenous antioxidant. This molecule affects the pentose phosphate pathway through the glutathione reductase enzyme, which generates NADP, as a result of the reduction of GSSG with NADPH + H^+^. In human RBCs, the reduced GSH amount displays a wide inter-individual variability from 0.4 to 3.0 mM. However, it has been widely demonstrated that different factors, including oxidative stress-related diseases, can alter reduced GSH levels (van 'T Erve et al., 2013; Bertelli et al., 2021; Ferrera et al., 2021; Remigante et al., 2023a). The data obtained confirmed that exposure to AAPH reduced (∼45%) GSH levels (Figure 9). The anthocyanin-enriched fraction exhibited a double response on the damaged RBCs. On one hand, the pre-incubation of human RBCs with the anthocyanin-enriched fraction completely restored GSH levels (Figure 9A). On the other hand, the post-incubation did not revert the effects of the oxidizing agent. However, these findings are in line with those reported in a former investigation (Lagana et al., 2020), which demonstrates that an anthocyanin-enriched fraction is able to scavenge different free radical species. This has been demonstrated via Electron Transfer and Hydrogen Atom Transfer reaction-based assays.

Antioxidant enzymes can work together to scavenge excessive ROS levels and maintain the redox balance in mature RBCs. For example, the SOD enzyme catalyses the dismutation of harmful O_2_^•−^ into O_2_ and H_2_O_2_. The H_2_O_2_ generated can then be decomposed into nontoxic H_2_O and O_2_ by means of other antioxidant enzymes such as catalase (CAT). In this regard, both SOD and CAT activity were investigated. The activity of both enzymes was much higher in RBCs incubated with AAPH than in untreated cells, which could reflect the activation of the endogenous antioxidant defense system to suppress the production of free radicals (Figures 9B,C). However, increased SOD and CAT activity failed to counterbalance the free radical rise, as demonstrated by the increase in lipid peroxidation levels as well as protein oxidation (Figures 5B,C). In this context, pre-exposure of RBCs to the anthocyanin-enriched fraction significantly prevented the upregulation in both SOD and CAT activity reported in AAPH-treated cells (Figures 9B,C). Both upregulation in CAT and SOD activity and concomitant substantial elevation of oxidative stress levels could reflect exhaustion of the endogenous antioxidant battery. These biochemical modifications may induce significant damage to the plasma membrane lipid components as well as protein structures. As a result, the membrane mechanical properties could be altered, resulting in a reduction of fluidity and deformability or altered permeability of the phospholipid bilayer, which, in turn, reduces the ability of the plasma membrane to withstand osmotic changes (Spinelli et al., 2023). These data indicate that anthocyanins might behave synergistically with the endogenous antioxidant machinery to counteract oxidative stress events in mature RBCs and preserve cellular integrity.

## 4 Materials and methods

### 4.1 Preparation of anthocyanin-enriched fraction of acidified ethanolic extract from *Callistemon citrinus* flowers

Flowers of *Callistemon citrinus* were collected from local nurseries in Messina (Italy), hair dried until they reached a moisture content lower than 2%, powdered with a mortar, and used for the extraction of anthocyanins. A total of 1 g of powder was extracted several times with a 1:10 (w/v) acetic acid:ethanol:water (1:70:29, v/v/v) mixture. The volume obtained was concentrated to 5.0 mL with a rotary evaporator, extracted again, and concentrated, followed by a solid phase extraction (SPE) by a Supelclean™ LC-18 SPE cartridge (Supelco Ltd., Bellefonte, PA, USA). The final elution has been performed with an acetic acid:ethanol:water (1:70:29, v/v/v) mixture. The anthocyanin-rich fraction was dried with a yield of ∼21%. The powder obtained was stored in the dark at 4°C.

### 4.2 Anthocyanin profile characterization by RP-HPLC-DAD

Qualitative and quantitative determination of anthocyanins was carried out using a Shimadzu system, consisting of an LC-10AD pump system, a vacuum degasser, a quaternary solvent mixing, an SPD- M10A diode array detector, and a Rheodyne 7725i injector. The chromatographic separation was carried out using a Luna Omega PS C18 column (150 × 2.1 mm, 5 μm; Phenomenex) with solvent A (formic acid 0.1%) and solvent B (acetonitrile), according to the following gradient elution program: 0–3 min, 0% B; 3–9 min, 3% B; 9–24 min, 12% B; 24–30 min, 20% B; 30–33 min, 20% B; 33–43 min, 30% B; 43–63 min, 50% B; 63–66 min, 50% B; 66–76 min, 60% B; 76–81 min, 60% B; 81–86 min, 0% B, and equilibrated 4 min for a total run time of 90 min. The flow rate, injection volume, and column temperature were 0.4 mL/min, 5 μL, and 25 °C, respectively. UV-visible spectra of anthocyanins were recorded between 220 and 800 nm wavelength and chromatograms were acquired at 260, 290, 330, 370, and 520 nm wavelength to detect the eventual presence of different polyphenols classes. The peak identity was confirmed by comparing retention times and UV-visible spectra with those reported in the literature and with authentic standards when commercially available. Due to the presence of poly-glycosylated and polymeric anthocyanins, the anthocyanin content was expressed as cyanidin-3-O-glucoside equivalents/100 g of dry extract (DE) by using an external calibration curve made with a reference standard.

### 4.3 Solutions and chemicals for RBC sample processing

All chemicals were purchased from Sigma (Milan, Italy). Regarding stock solutions, 4,4′-diisothiocyanatostilbene-2,2′-disulfonate (DIDS, 10 mM) and 2,2′-Azobis (2-methylpropionamidine) dihydrochloride (AAPH, 0.5 M) were dissolved in dimethyl sulfoxide (DMSO); N-ethylmaleimide (NEM, 310 mM) was dissolved in ethanol. H_2_O_2_ experimental solution was obtained by diluting a 30% w/v stock solution in distilled water. Both ethanol and DMSO never exceeded 0.001% v/v in the experimental solutions and were previously tested on RBCs to exclude possible haemolytic damage.

### 4.4 Preparation of human RBCs

The experiments were carried out on male (mean age 53 ± 5 years) and female (mean age 54 ± 5 years) non-smoking donors. Following the rules of good medical practice, the nature and purpose of the study were explained to all participants who then gave their informed consent. All donors had stopped taking aspirin or NSAIDs at least 1 week before the start of the study. To exclude the interference of sex hormones with the aggregation of red blood cells, only menopausal women who had not taken hormone replacement therapy were enrolled. Whole human blood was collected in test tubes containing ethylenediaminetetraacetic acid (EDTA). Red blood cells were washed in isotonic solution (NaCl 150 mM, 4-(2-hydroxyethyl)-1-piperazineethanesulfonic acid (HEPES) 5 mM, glucose 5 mM, pH 7.4, osmotic pressure 300 mOsm/kgH_2_O) and centrifuged (Neya 16R, 1,200×g, 5 min) to discard both plasma and buffy coat. Then, RBCs were suspended to different haematocrits in isotonic solution according to the experimental tests. The experimental design shown in Figure 2 was applied.

### 4.5 Analysis of cell shape by scanning electron microscopy (SEM)

RBCs samples were left untreated or exposed to a 50 mM AAPH-containing isotonic solution for 1 h at 37 °C with or without pre- or post-incubation with 0.01 μg/mL *Callistemon citrinus* extract for 1 h at 37°C. Next, RBCs were collected, plated on poly-l-lysine-coated slides, and fixed with 2.5% glutaraldehyde in 0.1 M cacodylate buffer (pH 7.4) at room temperature for 20 min. Then, samples were post-fixed with 1% OsO_4_ in 0.1 M sodium cacodylate buffer and dehydrated via a graded series of ethanol solutions from 30% to 100%. Then, absolute ethanol was gradually substituted by a 1:1 solution of hexamethyldisilazane (HMDS)/absolute ethanol and successively by pure HMDS. As a further step, HMDS was completely removed and samples were dried in a desiccator. Dried samples were mounted on stubs, coated with gold (10 nm), and analysed by a Cambridge 360 scanning electron microscopy (Leica Microsystem, Wetzlar, Germany), as formerly described (Straface et al., 2002). The number of RBCs with an altered shape (acanthocytes) was evaluated by counting ≥ 500 cells (50 RBCs for each different SEM field with a magnification of 3,000x) from samples in triplicate.

### 4.6 Determination of lactate dehydrogenase (LDH) release

To evaluate the amount of lactate dehydrogenase (LDH) released, the human RBCs were left untreated or treated with 50 mM AAPH for 1 h at 37 °C with or without pre- or post-treatment with the anthocyanin-enriched fraction (0.01 μg/mL) for 1 h at 37 °C. The RBCs were centrifuged at 1,200 *×g* for 10 min to save the supernatant. The latter was used for the determination of the amount of LDH released and was homogenized by sonication and centrifuged at 14,000xg for 10 min. The amount of LDH released has been quantified as lactate conversion to pyruvate using nicotinamide adenine dinucleotide (NAD^+^) as a hydride acceptor. LDH activity is directly proportional to the absorbance decrease at 340 nm wavelength. The amount of LDH released has been expressed as % of the maximum amount of LDH in an untreated sample.

### 4.7 Measurements of mean corpuscular volume (MCV)

Mean corpuscular volume (MCV) is the average volume of RBCs. It has been measured by automated haematology analyser (BC-6800 PLUS, Medical Systems, Milan, Italy) in left untreated RBCs or treated with 50 mM AAPH (1 h, at 37°C) with or without pre- or post-treatment with anthocyanin-enriched fraction (0.01 μg/mL) for 1 h, at 37°C. Such parameter was calculated from haematocrit and RBC count, as follows: MCV in fl=(Hct [in L/L]/RBC [in x10-12/L]) x 1,000.

### 4.8 Assessment of oxidative stress parameters

#### 4.8.1 Detection of reactive oxygen species (ROS) levels

To evaluate intracellular ROS content, RBCs, left untreated or exposed to AAPH-containing solution (for 1 h, at 37°C) with or without pre- or post-treatment with anthocyanin-enriched fraction (0.01 μg/mL, for 1 h, at 37°C) were incubated in Hanks’ balanced salt solution, pH 7.4, containing dihydrorhodamine 123 (DHR 123; Molecular Probes) and then analyzed with a FACScan flow cytometer (Becton-Dickinson, Mountain View, CA, USA). At least 20.000 events were acquired. The median values of fluorescence intensity histograms were used to provide a semi-quantitative analysis of ROS production (Lucantoni et al., 2006).

#### 4.8.2 Measurement of TBARS levels

Thiobarbituric-acid-reactive substances (TBARS) levels were measured as reported by Mendanha and collaborators (Mendanha et al., 2012). TBARS are derived from the reaction between thiobarbituric acid (TBA) and malondialdehyde (MDA), which is the end-product of lipid peroxidation. Red blood cells were incubated with 50 mM AAPH for 1 h at 37 °C with or without pre- or post-incubation with the anthocyanin extract (0.01 μg/mL) for 1 h at 37 °C. Successively, samples were centrifuged (Neya 16R, 1,200 *×g*, 5 min) and suspended in isotonic solution. Samples (1.5 mL) were treated with 10% (w/v) trichloroacetic acid (TCA) and centrifuged (Neya 16R, 3,000 *×g*, 10 min). TBA (1% in 0.5 mM NaOH hot solution, 1 mL) was added to the supernatant and the mixture was incubated at 95 °C for 30 min. At last, TBARS levels were obtained by subtracting 20% of the absorbance at 453 nm from the absorbance at 532 nm (Onda Spectrophotometer, UV-21). Results are indicated as µM TBARS levels (1.56 × 10^5^ M^−1^ cm^−1^ M extinction coefficient).

#### 4.8.3 Measurement of total -SH content

Measurement of total -SH groups was carried out according to the method of Aksenov and Markesbery (Aksenov and Markesbery, 2001). In short, RBCs were left untreated or exposed to an isotonic AAPH-containing solution for 1 h at 37 °C with or without pre- or post incubation with the anthocyanin extract (0.01 μg/mL) for 1 h at 37 °C). Next, RBCs were centrifuged (Neya 16R, 1,200 *×g*, 5 min) and a 100 µL sample was haemolysed in 1 mL of distilled water. A 50 μL aliquot of the haemolysis product was added to 1 mL of phosphate-buffered saline (PBS, pH 7.4) containing EDTA (1 mM). Then, the addition of 5,5′-Dithiobis (2-nitrobenzoic acid) (DTNB, 10 mM, 30 μL) initiated the reaction and the samples were incubated for 30 min at 25°C protected from light. Control samples without cell lysate or DTNB were processed in parallel. After incubation, sample absorbance was measured at 412 nm (Onda spectrophotometer, UV-21) and 3-thio-2-nitro-benzoic acid (TNB) levels were detected after subtraction of blank absorbance determined on samples containing only DTNB. To achieve full oxidation of -SH groups as the positive control, an aliquot of RBCs was incubated with 2 mM NEM for 1 h at 25 °C (Morabito et al., 2015; Morabito et al., 2016). Data were normalised to protein content and results reported as μM TNB/mg protein.

#### 4.8.4 Measurement of methaemoglobin (MetHb) levels

The MetHb content was determined as reported by Naoum and collaborators (Naoum and Magaly Da Silva, 2004). This assay is based on MetHb and (oxy)-hemoglobin (Hb) determination by spectrophotometry at 630 and 540 nm wavelength, respectively. After incubation with 50 mM AAPH for 1 h at 37 °C with or without pre- or post-treatment with the anthocyanin extract (0.01 μg/mL) for 1 h at 37°C, 25 μL of RBCs resuspended were lysed in 1975 μL hypotonic buffer (2.5 mM NaH_2_PO_4_, pH 7.4, 4°C). Then, samples were centrifuged (13,000 *×g*, 15 min, 4°C; Eppendorf) to remove plasma membranes and the absorbance of the supernatant was measured (BioPhotometer Plus; Eppendorf). To induce a complete haemoglobin (Hb) oxidation, a sample of RBCs was incubated for 1 h at 25 °C with 4 mM NaNO_2_, a well-known MetHb-forming compound. The percentage (%) of MetHb was determined as follows: % MetHb= (OD 630/OD 540) × 100 (OD), where OD is the optical density.

### 4.9 Preparation of RBC membranes

Cell plasma membranes were processed as described by Pantaleo and collaborators (Pantaleo et al., 2016). Blood samples were suspended in hypotonic cold solution (2.5 mM NaH_2_PO_4_) containing a protease inhibitor mixture and were centrifuged (Eppendorf, 4 °C, 18,000 *×g*, 10 min) to discard haemoglobin. Plasma membranes were then solubilized with SDS (1% v/v) and put on ice for 20 min. The supernatant containing the solubilized plasma membrane proteins was finally conserved at −80 °C.

#### 4.9.1 SDS-PAGE preparation and western blotting analysis

Red blood cell plasma membranes were heated for 10 min at 95 °C after dissolving in Laemmli buffer (Laemmli, 1970). The proteins were separated using SDS-polyacrylamide gel electrophoresis and transferred to a polyvinylidene fluoride membrane by maintaining a constant voltage for 2 h. Membranes were blocked for 1 h at 25 °C in BSA and incubated at 4°C with the primary antibodies diluted in TBST (mouse monoclonal anti-band 3, B9277, Sigma-Aldrich, Milan, Italy, 1:1,000; mouse monoclonal anti-P-TyR antibody (tyrosine), T1325, Sigma-Aldrich, Milan, Italy, 1:1,000; and rabbit monoclonal anti-Syk, SAB4500552, Sigma-Aldrich, Milan, Italy, 1:500). Successively, membranes were incubated with peroxidase-conjugated goat anti-mouse/rabbit IgG secondary antibodies (A9044/A0545, Sigma-Aldrich, Milan, Italy) diluted 1:10,000/1:20,000 in TBST solution for 1 h at 25 °C. To quantify the protein in equal amounts, a mouse monoclonal anti-GAPDH antibody (SC-32233, Santa Cruz Biotechnology, Italy, 1:10,000 in TBST), was incubated with the same membrane, as reported by Yeung and co-authors (Yeung and Stanley, 2009). A system of chemiluminescence detection (Super Signal West Pico Chemiluminescent Substrate, Pierce Thermo Scientific, Rockford, IL, USA) was employed to obtain the signal for image analysis (Image Quant TL, v2003). The intensity of protein bands was determined by densitometry (Bio-Rad ChemiDocTM XRS+).

### 4.10 Analytical cytology

Red blood cells were left untreated or exposed to an AAPH-containing isotonic solution for 1 h at 37 °C with or without pre- or post-incubation with the anthocyanin extract (0.01 μg/mL) for 1 h at 37 °C). Next, RBCs were fixed with 3.7% formaldehyde in PBS (pH 7.4) for 10 min at room temperature and then washed in the same buffer. Cells were then permeabilized with 0.5% Triton X-100 in PBS for 5 min at room temperature. After washing with PBS, samples were incubated with a mouse monoclonal anti-band 3 antibody (Sigma, Milan, Italy) for 30 min at 37°C, washed, and then incubated with a fluorescein isothiocyanate (FITC)-labeled anti-mouse antibody (Sigma, Milan, Italy) for 30 min at 37°C (Giovannetti et al., 2012). Cells incubated with the secondary antibody given alone were used as the negative control. Samples were analyzed by an Olympus BX51 Microphot fluorescence microscope or by a FACScan flow cytometer (Becton Dickinson, Mountain View, CA, USA) equipped with a 488 nm argon laser. At least 20,000 events have been acquired. The median values of fluorescence intensity histograms are given to provide a semiquantitative analysis. Fluorescence intensity values were normalised for those of untreated erythrocytes and expressed in %.

### 4.11 Measurement of SO_4_^2−^ uptake

#### 4.11.1 Control condition

The anion exchange through band 3 was determined as the uptake of SO_4_^2−^ in RBCs, as described elsewhere (Romano and Passow, 1984; Romano et al., 1998; Morabito et al., 2018; Morabito et al., 2019b). Briefly, after washing, RBCs were suspended in 35 mL SO_4_^2−^ medium (composition in mM: Na_2_SO_4_ 118, HEPES 10, glucose 5, pH 7.4, osmotic pressure 300 mOsm/kgH_2_O) and incubated at 25 °C for 5, 10, 15, 30, 45, 60, 90, and 120 min. After each incubation time, DIDS (10 μM), which is an inhibitor of band 3 activity (Jessen et al., 1986), was added to 5 mL sample aliquots, which were kept on ice. To eliminate SO_4_^2−^ from the external medium, samples were washed three times in cold isotonic solution and centrifuged (Neya 16R, 4 °C, 1,200 *×g*, 5 min). Distilled water was added to the cell pellet to induce osmotic lysis and perchloric acid (4% v/v) was used to precipitate proteins. After centrifugation (Neya 16R, 4 °C, 2,500 *×g*, 10 min), the supernatant, which contained SO_4_^2−^ trapped by erythrocytes during the fixed incubation times, was subjected to the turbidimetric analysis. To this end, 500 μL of the supernatant were sequentially mixed to 500 μL glycerol diluted in distilled water (1:1), 1 mL 4 M NaCl, and 500 μL 1.24 M BaCl_2_•2H_2_O. Finally, the absorbance of each sample was measured at 425 nm (Spectrophotometer, UV-21, Onda Spectrophotometer, Carpi, Modena, Italy). A calibrated standard curve, which was obtained in a separate experimental set by precipitating known SO_4_^2−^ concentrations, was used to convert the absorbance into [SO_4_^2−^] L cells × 10^–2^. The rate constant of SO_4_^2−^ uptake (min^−1^) was derived from the following equation: C_t_ = C_∞_ (1 − e^−rt^) + C_0_, where C_t_, C_∞_, and C_0_ indicate the intracellular SO_4_^2−^ concentrations measured at time t, ∞, and 0, respectively, e represents the Neper number (2.7182818), r indicates the rate constant of the process, and t is the specific time at which the SO_4_^2−^ concentration was measured. The rate constant is the inverse of the time needed to reach ∼63% of total SO_4_^2−^ intracellular concentration (Romano et al., 1998). Results are reported as [SO_4_^2−^] L cells × 10^–2^ and represent the SO_4_^2−^ micromolar concentration internalized by 5 mL erythrocytes suspended to 3% haematocrit.

#### 4.11.2 Experimental conditions

After treatment with AAPH (50 mM) for 1 h at 37 °C proceeded or followed by 1 h incubation with or without the anthocyanin extract (0.01 μg/mL at 37°C), RBCs (3% haematocrit) were centrifuged (Neya 16R, 4°C, 1,200 *×g*, 5 min) and suspended in the SO_4_^2−^ -containing supernatant. The rate constant of SO_4_^2−^ uptake was then determined as described for the control condition.

### 4.12 Preparation of resealed ghosts and SO_4_^2−^ uptake measurement

Resealed ghosts were prepared from human RBCs as described by Morabito and co-authors (Morabito et al., 2016; Morabito et al., 2020). In short, RBC samples were washed, suspended in 35 mL of cold hypo-osmotic buffer (NaH_2_PO_4_ 2.5 mM, HEPES 5 mM, pH 7.4), and incubated for 10 min at 0 °C. Successively, the intracellular haemoglobin was discarded by multiple centrifugations (Neya 16R, 4 °C, 13.000 *×g*, 30 min). The supernatant was replaced by 35 mL of isotonic resealing medium (NaCl 145 mM, HEPES 5 mM, glucose 5 mM, pH 7.4, 300 mOsm/kg_H2O_, 37°C). The plasma membranes were then incubated for 45 min at 37°C to allow for the correct resealing. Finally, resealed ghosts, which contained ∼10% of the total haemoglobin, were treated with AAPH (50 mM) for 1 h at 37°C preceded or followed by incubation with or without the anthocyanin-enriched fraction (0.01 μg/mL) for 1 h at 37°C. The SO_4_^2-^ uptake was measured according to the protocol described above for intact RBCs.

### 4.13 Assessment of the Endogenous Antioxidant Activity

#### 4.13.1 Determination of reduced glutathione (GSH) levels

After treatment with 50 mM AAPH for 1 h at 37 °C with or without pre- or post-treatment with the anthocyanin-enriched fraction (0.01 μg/mL for 1 h at 37 °C), RBC samples were centrifuged at 1,200 *xg* for 10 min to discard the supernatant. Each sample was homogenized by sonication and an aliquot was withdrawn and diluted with an equal volume of 5-sulfosalicylic acid solution (5%) to accomplish sample deproteinization. Samples were then centrifuged at 13,000 *xg* for 10 min. Intracellular reduced glutathione (GSH) levels were assayed according to Alisik and collaborators (Alisik et al., 2019). The quantification was performed by a standard curve obtained with pure GSH. Results are indicated as mM GSH. Both AAPH and the anthocyanin-enriched fraction did not interfere with the determination of GSH levels.

#### 4.13.2 Catalase (CAT) activity assay

Catalase (CAT) activity was evaluated by the catalase assay kit (MAK381, Sigma-Aldrich, Milan, Italy), according to the manufacturer´s instructions. RBCs were exposed to 50 mM AAPH for 1 h at 37°C with or without pre- or post-incubation with the anthocyanin extract (0.01 μg/mL) for 1 h at 37 °C. As the positive control, cells were exposed to 20 mM H_2_O_2_ for 30 min. After each treatment, the incubation medium was discarded and cells were washed in PBS 1X. Subsequently, cells were lysed in 0.2 mL catalase assay buffer and centrifuged at 10,000 for 15 min at 4 °C. The supernatant (50 µL) was incubated with 12 µL of 20 mM H_2_O_2_ for 30 min at 25 °C. Stop solution (10 µL) and developer reaction mix (50 µL) were added and the samples were incubated for 10 min at 25 °C. CAT activity was determined by reading the absorbance at 570 nm wavelength (Fluostar Omega, BMG Labtech, Ortenberg, Germany) after subtracting the background.

#### 4.13.3 Superoxide dismutase (SOD) activity assay

Superoxide dismutase (SOD) activity was evaluated by a specific assay (CS0009, Sigma-Aldrich, Milan, Italy), according to the manufacturer´s instructions. RBCs were exposed to 50 mM AAPH for 1 h at 37 °C with or without pre- or post-incubation with the anthocyanin extract (0.01 μg/mL) for 1 h at 37°C. As the positive control, cells were exposed to 20 mM H_2_O_2_ for 30 min. Subsequently, cells were lysed in 1:5 distilled water. The lysate was collected in 0.5 mL tubes and centrifuged at 10,000 *×g* for 10 min at 4°C. The supernatant (20 µL) was transferred to a 96-well plate. Then, 20 µL dilution buffer, 160 µL WST solution and 20 µL xanthine oxidase solution were sequentially added. The samples were incubated for 1 h at 25 °C and SOD activity was determined by reading the absorbance at 450 nm wavelength (Fluostar Omega, BMG Labtech, Ortenberg, Germany) after subtracting the background.

### 4.14 Experimental data and statistics

All data are expressed as arithmetic mean ± SEM. For statistical analysis and graphics, GraphPad Prism (version 8.0, GraphPad Software, San Diego, CA, USA) and Excel (Version 2019; Microsoft, Redmond, WA, USA) software were used. Significant differences between mean values were determined by one-way analysis of variance (ANOVA) followed by Bonferroni’s post-hoc test. Statistically significant differences were assumed at *p* < 0.05; (n) corresponds to the number of independent measurements.

## 5 Conclusion

In conclusion, exposure of RBCs to AAPH increased oxidative stress levels, resulting in morphological alterations, namely, the formation of acanthocytes, increased lipid peroxidation and oxidation of proteins, as well as abnormal distribution and hyper-phosphorylation of band 3. Expected, AAPH incubation was also associated with a decrease of the band 3 functional activity and an increased amount of oxidized haemoglobin, which led to abnormal clustering of band 3. Finally, exposure to AAPH provoked both the consumption of reduced GSH and the over-activation of the endogenous antioxidant machinery, represented by catalase and superoxide dismutase. Pre-treatment of RBCs with an anthocyanin-enriched fraction effectively prevented all these oxidative stress-related alterations to a significant extent. In contrast, exposure of RBCs to the antioxidant compounds after the oxidative insult was less effective. These results reveal that most oxidative stress-associated modifications in RBCs are irreversible, but early implementation of natural antioxidant compounds can prevent or neutralize oxidative stress.

Indeed, exposure of RBCs to the anthocyanin fraction prior to, but not after, oxidative stress could protect band 3 function from oxidative stress-dependent inhibition. However, this effect can only be observed in intact RBCs but not in RBC ghosts, pointing to the fact that cellular integrity and preservation of a cytosolic component are essential prerequisites for the prevention of oxidative stress-related functional alterations in RBCs. In addition, the present study provides mechanistic insights into the different benefits deriving from the use of naturally occurring anthocyanins against oxidative stress on a cellular level. Taking into account that oxidative stress is involved in a large number of pathologies, new biomarkers of oxidative stress with diagnostic and monitoring potential are needed. In this regard, blood can be obtained from patients with minimally-invasive procedures, reflects the physiological states of peripheral tissues and cells, and could be considered as a remarkable source of oxidative stress biomarkers. In particular, we propose band 3 (SLC4A1/AE1) activity as a novel oxidative stress biomarker. Further studies (*ex vivo*) are needed to clarify the mechanisms underlying the oxidative stress events on RBC homeostasis, including potential effects on the interaction between band 3 and the cytoskeletal proteins ankyrin and spectrin, their possible post-translation modifications, as well as the mechanisms by which metabolism of RBCs regulates the transport systems of gas exchange.

## Data Availability

The original contributions presented in the study are included in the article/Supplementary Materials, further inquiries can be directed to the corresponding author.
