# Supplementary material for: AAPH-induced oxidative damage reduced anion exchanger 1 (SLC4A1/AE1) activity in human red blood cells: protective effect of an anthocyanin-rich extract
Source: Front Physiol. 2023 Dec 4;14:1303815. doi: 10.3389/fphys.2023.1303815 (PMC10725977; doi:10.3389/fphys.2023.1303815)

**Supplementary Materials**

**1. Results**

**Figure S1. Effect of increasing concentrations of the anthocyanin-enriched fraction on haemolysis, TBARS levels, and sulfhydryl groups total content.** In A, B and C, haemolysis measurement is shown. Red blood cells were incubated with increasing concentrations of the anthocyanin-enriched fraction for 30 min, or alternatively, for 1 h and 2 h at 37 °C. Complete haemolysis was obtained with distilled water (dH_2_O). ns, not statistically significant versus untreated; ***p<0.001 versus untreated, ANOVA with Dunnet’s post-test (n=12). In D, E, and F, oxidative stress assessment by estimation of TBARS levels is shown. Red blood cells were incubated with increasing concentrations of the anthocyanin-enriched fraction for 30 min, 1 h, and 2 h at 37° C or with 20 mM H_2_O_2_ as a positive control. ns, not statistically significant versus untreated; *p<0.05 and **p<0.001 versus untreated, ANOVA with Dunnet’s post-test (n=12). In G, H and I, oxidative stress assessment by estimation of total sulfhydryl group content (µM TNB/µg protein) is shown. Red blood cells were incubated with increasing concentrations of the anthocyanin-enriched fraction for 30 min, or alternatively, for 1 h and 2 h at 37 °C or with 2 mM NEM as a positive control. ns, not statistically significant versus untreated; *** p<0.001 versus untreated, ANOVA with Dunnet’s post-test (n=12).

Figure S2. Apoptosis evaluation by flow cytometry. Percentage of apoptotic RBCs, namely positive to annexin 5 and trypan blue, detected after incubation with 50 AAPH (for 1 h, at 37 °C), with or without exposure to the anthocyanin-enriched fraction (0.01 μg/mL for 1 h, at 37 °C). ns, not statistically significant versus untreated cells, ANOVA with Dunnet’s post-test (n=10).

Original Blots

Band 3 protein (AE1)


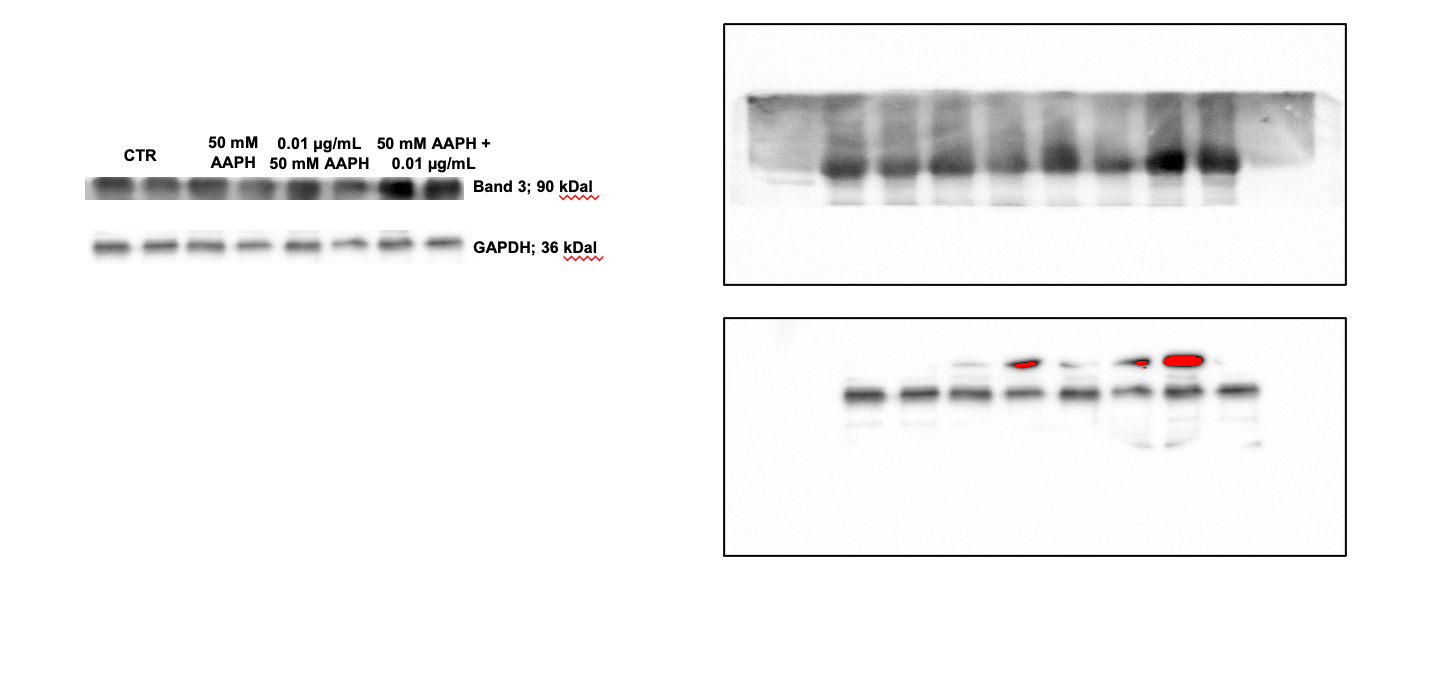


SyK kinase


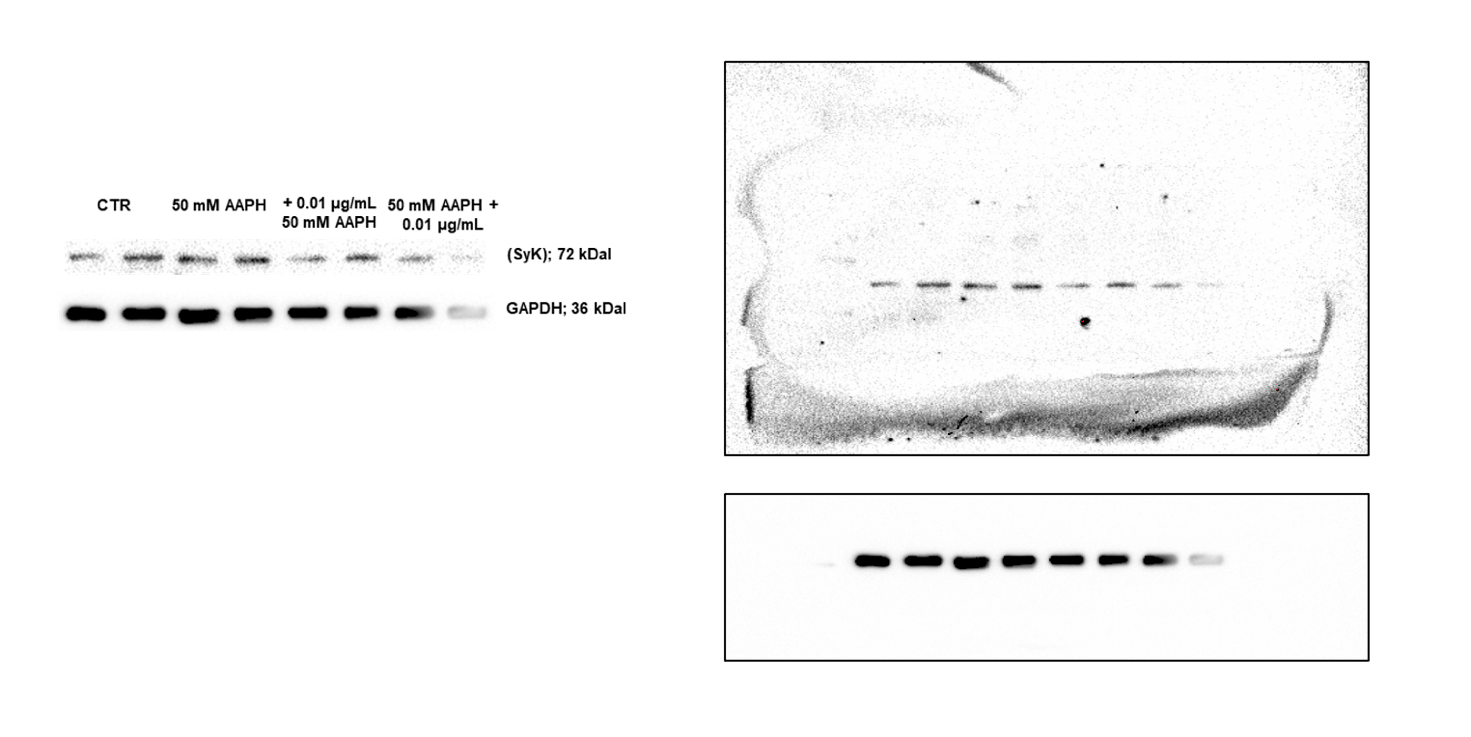


p-TyR Band 3 protein


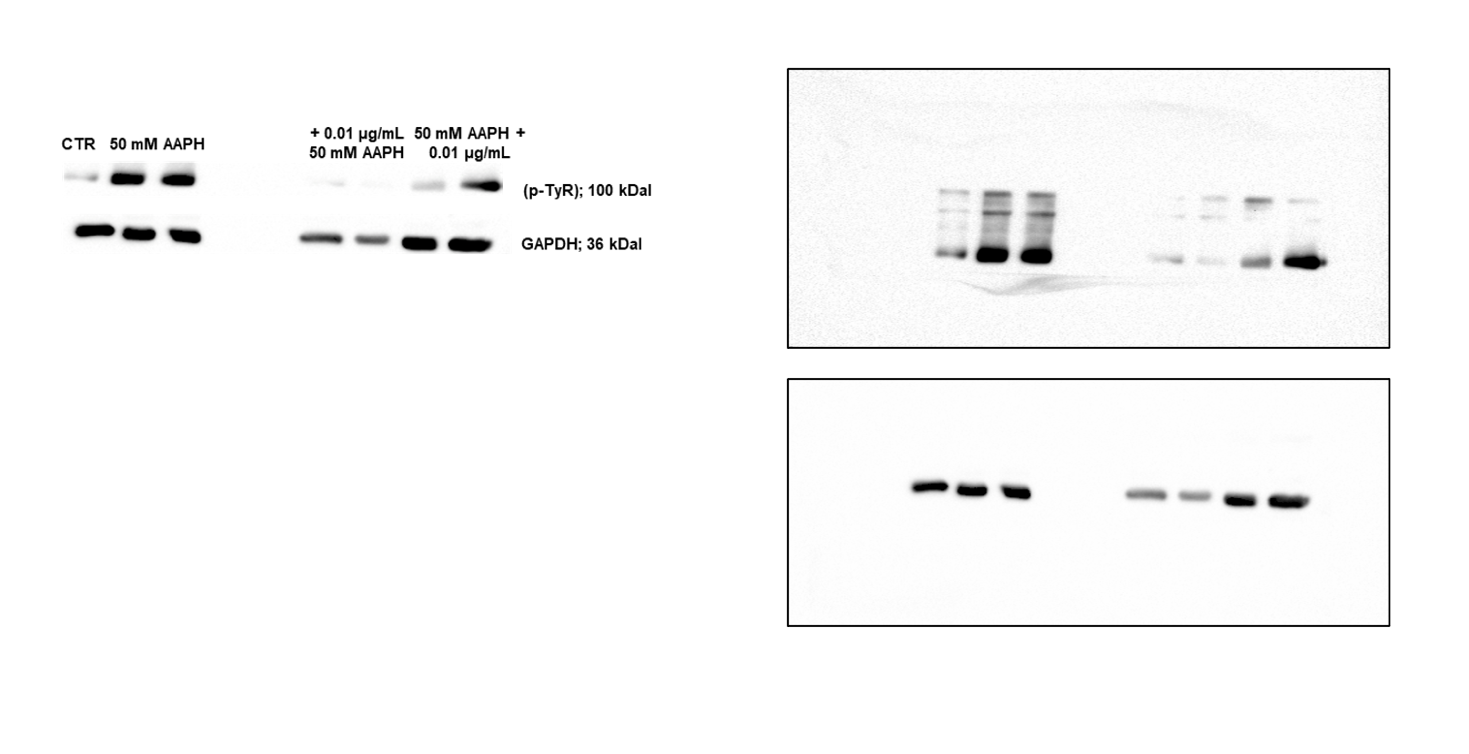

Supplement: Supplementary file 1 [file Table1.DOCX]
